# Metrological evaluation of DNA extraction method effects on the bacterial microbiome and resistome in sputum

**DOI:** 10.1128/msystems.00735-24

**Published:** 2024-08-16

**Authors:** Aleksander Benčič, Nataša Toplak, Simon Koren, Alexandra Bogožalec Košir, Mojca Milavec, Viktorija Tomič, Dane Lužnik, Tanja Dreo

**Affiliations:** ^1^Department of Biotechnology and Systems Biology, National Institute of Biology, Ljubljana, Slovenia; 2 Jožef Stefan International Postgraduate School, Ljubljana, Slovenia; 3 OMEGA d.o.o., Ljubljana, Slovenia; 4 University Clinic of Pulmonary and Allergic Diseases Golnik, Laboratory for Respiratory Microbiology, Golnik, Slovenia; Cleveland Clinic, Cleveland, Ohio, USA

**Keywords:** targeted high-throughput sequencing, bacterial microbiome, resistome, bacteria detection, DNA extraction

## Abstract

Targeted high-throughput sequencing (HTS) has revolutionized the way we look at bacterial communities. It can be used for the species-specific detection of bacteria as well as for the determination of the microbiome and resistome and can be applied to samples from almost any environment. However, the results of targeted HTS can be influenced by many factors, which poses a major challenge for its use in clinical diagnostics. In this study, we investigated the impact of the DNA extraction method on the determination of the bacterial microbiome and resistome by targeted HTS using principles from metrology and diagnostics such as repeatability and analytical sensitivity. Sputum samples spiked with *Acinetobacter baumannii*, *Klebsiella pneumoniae*, and *Pseudomonas aeruginosa* at three different concentrations (10^3^–10^6^ cells/mL) were used. DNA was extracted from each sample on 2 separate days in three replicates each using three different extraction methods based on cetrimonium bromide, magnetic beads, and silica membranes. All three spiked bacteria were detected in sputum, and the DNA extraction method had no significant effect on detection. However, the DNA extraction method had significant effects on the composition of the microbiome and the resistome. The sequencing results were repeatable in the majority of cases. The silica membrane-based DNA extraction kit provided the most repeatable results and the highest diversity of the microbiome and resistome. Targeted HTS has been shown to be a reliable tool for determining the microbiome and resistome; however, the method of DNA extraction should be carefully selected to minimize its impact on the results.

**IMPORTANCE:**

High-throughput sequencing (HTS) is one of the crucial new technologies that gives us insights into previously hidden parts of microbial communities. The DNA extraction method is an important step that can have a major impact on the results, and understanding this impact is of paramount importance for their reliable interpretation. Our results are of great value for the interpretation of sputum microbiome and resistome results obtained by targeted HTS. Our findings allow for a more rational design of future microbiome studies, which would lead to higher repeatability of results and easier comparison between different laboratories. This could also facilitate the introduction of targeted HTS in clinical microbiology for reliable identification of pathogenic bacteria and testing for antimicrobial resistance (AMR). As AMR is a major threat to public health, the improved methods for determining AMR would bring great benefits to both the healthcare system and society as a whole.

## INTRODUCTION

The microbiome of the lower respiratory tract plays an important role in the development and progression of lung diseases and the development of bacterial infections (1–3). Genes associated with antimicrobial resistance (AMR) are also part of the lung microbiome and define the so-called “resistome.” The term “resistome” is similar to the term “microbiome” and includes all genes associated with AMR. The role of the lung resistome is not yet well understood. The AMR genes in bacteria that make up the microbiota of healthy lungs form the core resistome, which is also a reservoir from which pathogenic bacteria can acquire AMR (4, 5).

Over the last decade, enormous progress has been made in microbiome and resistome research through the development of high-throughput sequencing (HTS) methods. These have enabled culture-independent microbiome analysis, in particular by targeted sequencing of the hypervariable regions of 16S ribosomal RNA (rRNA) (6, 7). Similarly, targeted HTS can also be used to detect genetic elements associated with AMR or horizontal gene transfer of such elements (8–10). However, when studying the lung microbiome and resistome, there can be variability in each step of the required procedures. These include sample collection, storage, DNA extraction, PCR amplification, library preparation, sequencing, and bioinformatic analysis (11, 12). DNA extraction has been shown to be one of the main sources of variability and bias in microbiome analysis and is therefore a critical step in the process. Therefore, the choice of DNA extraction method is an important part of the design of any study to determine the microbiome and resistome (7, 11, 13–17).

Sputum is an attractive source material for lung microbiome studies as it can be collected relatively easily and non-invasively (18). However, it is also a complex and heterogeneous matrix. The vast majority of DNA isolated from sputum is host DNA. To overcome these challenges, various methods for sample preparation and DNA extraction have been developed. Generally, solubilizers are used prior to DNA extraction from sputum to reduce differences between samples and increase DNA yield (19). Mechanical, enzymatic, and chemical methods can be used to ensure adequate cell lysis (16). Different approaches to sample preparation and DNA extraction can lead to differences in the determined composition of the microbiome. This also complicates the comparison of results from different studies and hinders a possible meta-analysis of these data. The comparison of different DNA extraction methods is further complicated by the fact that the actual composition of the microbial community is not known and it is also not known which DNA extraction method provides results that are closest to the actual state (20). To date, there are only a limited number of studies that have addressed the determination of the lung microbiome and resistome using sputum and there is a general lack of data on the effects of different DNA extraction methods on the composition (16). To facilitate the adoption of HTS in clinical diagnostics of respiratory diseases, it is of great importance that possible impacts of DNA extraction methods are identified and most suitable methods are recognized and implemented in practice.

**Fig 1:**
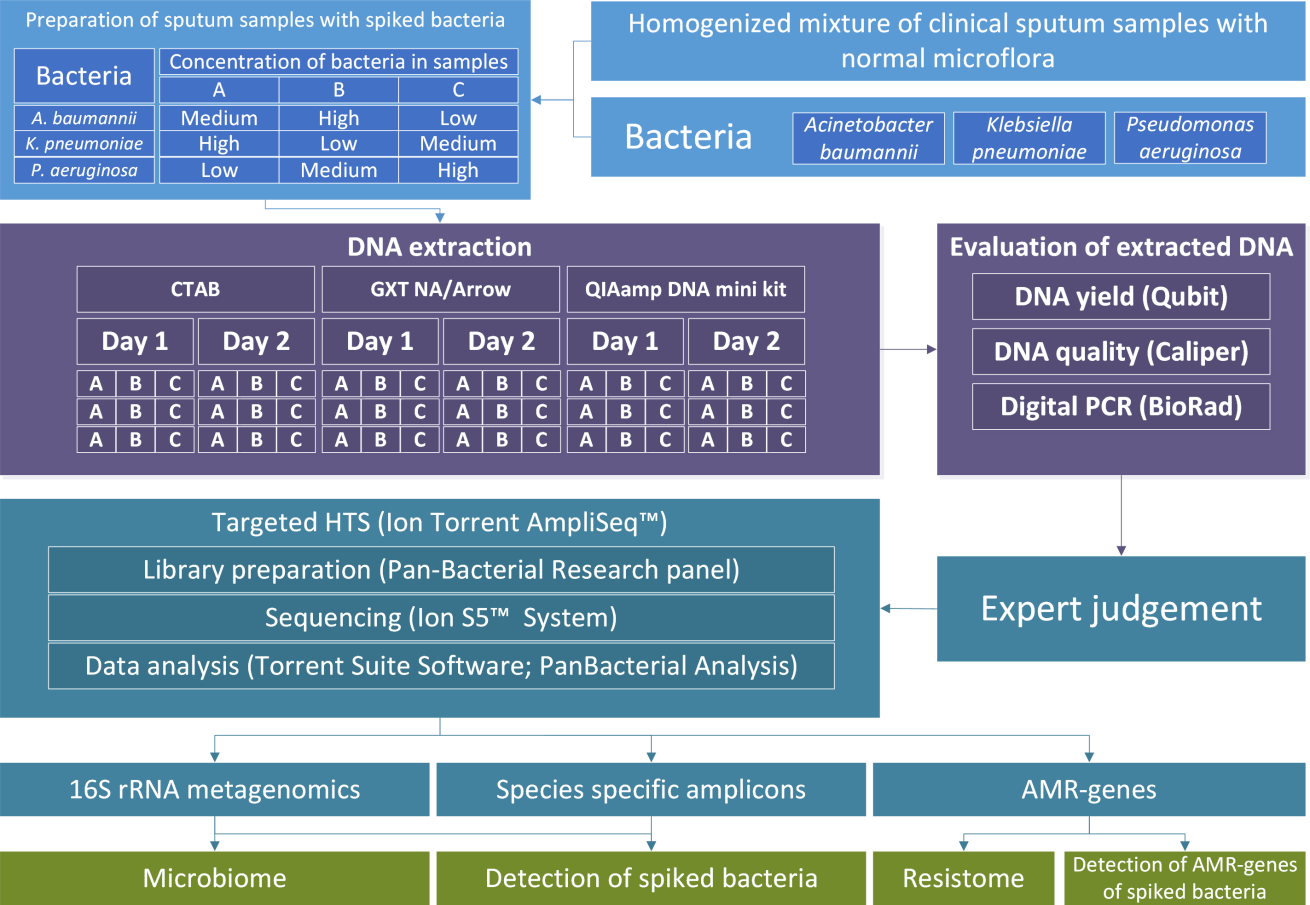
Scheme of the study design describing samples A, B, and C preparation from homogenized sputum samples and bacterial suspensions. DNA was extracted using three different DNA extraction methods on 2 separate days in three technical repeats. DNA yield and quality were analyzed prior to library preparation. From data obtained by targeted HTS, we detected spiked bacteria and AMR genes belonging to these bacteria, and determined microbiome and resistome.

The main objective of the present study was to investigate the impact of three DNA extraction methods on the results of targeted HTS and their repeatability and reproducibility from sputum samples. To this end, the principles of metrology and diagnostics were used to evaluate the results. Spiked sputum samples were used from which DNA was extracted on 2 different days in three replicates to perform targeted HTS. For DNA extraction, the following methods were used: an in-house cetrimonium bromide (CTAB) solution-based extraction and two commercially available kits for solid phase extraction, one based on magnetic beads and an extraction robot (GXT NA/Arrow), and the other on silica membranes (QIAamp DNA mini kit). Targeted HTS was used to determine the microbiome based on 16S rRNA, to determine the resistome, and for species-specific detection of bacteria. The target bacteria were *Acinetobacter baumannii*, *Klebsiella pneumoniae*, and *Pseudomonas aeruginosa* (Fig. 1). The repeatability of the DNA extraction methods was determined as the coefficient of variation (CV; %) of different quantities for the samples from which the DNA was extracted on the same day using the same extraction method.

## MATERIALS AND METHODS

### Sample preparation

The samples used in this study were prepared as part of the study conducted by Bogožalec Košir et al. (21). The sputum samples were collected from patients with lung diseases at the University Clinic of Respiratory and Allergic Diseases (Golnik, Slovenia). All sputum samples tested negative for the bacteria *A. baumannii*, *K. pneumoniae*, and *P. aeruginosa* with culture-based methods used in standard clinical diagnostics. In total, 16 sputum samples containing normal mixed lung microflora were pooled and digested with mucolytic reagent (1:1, vol/vol; Liquillizer; MetaSystems Hard & Software GmbH, Altlussheim, Germany). Bacterial suspensions were prepared from three bacteria: *A. baumannii* (DSMZ 30007), *K. pneumoniae* (DSMZ 30104), and *P. aeruginosa* (DSMZ 50071), which were obtained from the Deutsche Sammlung von Mikroorganismen und Zellkulturen (German Collection of Microorganisms and Cell Cultures). Spiked sputum samples were prepared by mixing aliquots of pooled and digested sputum with bacterial suspensions. Based on turbidity measurement, samples A, B, and C were prepared as randomized low, medium, and high concentrations (5.5 × 10^3^, 5.5 × 10^4^, and 5.5 × 10^5^ cells/mL, respectively) of *A. baumannii*, *K. pneumoniae*, and *P. aeruginosa* (Fig. 1; Table S1). The concentrations of the bacteria in the suspensions and the spiked sputum samples were additionally determined by direct digital PCR (without DNA extraction) (see “Digital PCR” below). These were the assigned concentrations of the three bacteria in the range of 10^3^ to 10^6^ cells/mL, which were used in the subsequent analysis (Table S2). A detailed description of samples preparation and DNA extraction is in Fig. S1 and S2, respectively.

### DNA extraction methods

DNA was extracted and purified from each spiked sample (200 µL) in three technical replicates on 2 separate days using the three different DNA extraction protocols. Negative controls (200 µL molecular grade water) were also extracted for each DNA extraction method on both extraction days. DNA extraction from the sample was performed as described by Bogožalec Košir et al. (21). The first method was a CTAB-based DNA extraction protocol adapted from Devonshire et al. (22). Prior to extraction, lysozyme (10 µL at 50 mg/mL) was added to each sample and incubated overnight at 37°C. At the end of the extraction, the pellets were rehydrated in 200 µL Tris-EDTA buffer overnight at 4°C and stored at −20°C. The second method was the GXT NA (Hain Lifescience GmbH, Tübingen, Germany) kit, which was used in combination with an automated nucleic acid extraction system (Arrow; NorDiag ASA, Bergen, Norway). DNA was eluted in 100 µL, according to the manufacturer’s instructions. The third method was the QIAamp DNA mini kit (Qiagen, Hilden, Germany). The DNA was eluted from the spin columns with 100 µL Buffer AE (included in the kits).

#### DNA extraction from bacterial suspensions

DNA was also extracted from pure bacterial suspensions of *A. baumannii*, *K. pneumoniae*, and *P. aeruginosa* using QIAamp DNA mini kit extraction protocol as described by Bogožalec Košir et al. (21). One sample (100 µL, 10^8^ cells/mL) of the suspension was extracted from each bacterium.

### DNA yield, concentration, and quality

Qubit dsDNA HS assay kits and a fluorimeter (Qubit 3.0; both Invitrogen, Carlsbad, CA, USA) were used to estimate DNA extraction yields (micrograms) and to measure the concentration of extracted DNA (nanograms per microliter). The quality of the extracted DNA was determined by its fragmentation profile using the Genomic DNA analysis on the LabChip GX capillary gel electrophoresis instrument (PerkinElmer, Inc., Hopkinton, MA, USA) and capillary gel electrophoresis. DNA fragmentation is expressed as a genomic quality score (GQS) ranging from 0 to 5, with lower values corresponding to higher DNA fragmentation.

### Digital PCR

Digital PCR was used to determine the bacterial concentration in the suspension prior to extraction and to determine the bacterial DNA concentration in extracts of sputum samples. The dPCR and subsequent data analysis were performed as described by Bogožalec-Košir et al. (21). The sequences of the primers and probes used for dPCR are listed in the Table S3. The dPCR experiments were performed on QX100/QX200 platform (BioRad). The dPCR mixtures had a total volume of 20 µL, which included 10 µL ddPCR supermix for probes (no dUTP), 6 µL primers and probe mix, and 4 µL DNA sample or bacterial suspension. Amplification conditions were 10 min DNA polymerase activation at 95°C, followed by 40 cycles of a two-step thermal profile of 30 s at 94°C for denaturation, and 60 s at 60°C for annealing and extension, followed by 10 min at 98°C, and then cooling to 4°C. Data were analyzed using the software package provided with the dPCR system (QuantaSoft 1.7.4.0917; BioRad) and Microsoft Excel. Reactions with droplet counts <8,000 per 20 µL PCR were excluded. Each suspension was tested in quadruplicate. Each dPCR run included a non-template control (water) and a positive control for each of the assays (synthetic DNA fragment with amplicon sequence for each assay).

### Targeted high-throughput sequencing

Targeted HTS was performed with an Ion Torrent platform (Thermo Fisher Scientific, Waltham, MA, USA) using ion semiconductor chemistry. The Ion AmpliSeq Pan-Bacterial Research Panel (Thermo Fisher Scientific), a community panel containing two primer pools for library construction, was used. The first pool consists of primer sets that enable species-specific detection of bacterial species and primers that target genes associated with antimicrobial resistance. The second pool consists of primers for the amplification of 16S rRNA regions.

#### Species-specific and antibiotic resistance amplicons

The first pool of the Pan-Bacterial Research Panel contains primers for 269 amplicons for the specific detection of 21 microbial species (Table S4). These include *A. baumannii*, *K. pneumoniae*, and *P. aeruginosa*, as used here. The other 18 microorganism species included are also known as possible pathogens. In addition, the first pool also contains primers for 716 amplicons belonging to 364 AMR genes associated with resistance to 31 different classes of antibiotics.

#### 16S rRNA sequencing

The second pool of the Pan-Bacterial Research Panel contains 24 amplicons for 16S rRNA profiling, which are arranged along the entire sequence of the 16S rRNA and designed in such a way that as many different bacteria as possible can be detected. The determination of the operational taxonomic units (OTUs) is based on approximately 400,000 16S rRNA sequences from the public Greengenes database, to which the reads are mapped after sequencing.

#### Library preparation

The Ion AmpliSeq Library Kit 2.0 (Thermo Fisher Scientific) was used to prepare the libraries using 10 ng of DNA extracted from the sputum samples. In cases where the DNA was too diluted to provide 10 ng, the maximum possible volume of DNA was added (3 µL). This was only the case for the negative controls of the DNA extraction and the positive controls (DNA extracted from bacterial suspensions). In addition, a negative control without template was also used for amplification in order to exclude possible contamination during PCR amplification. The composition of the PCR mixture for the 10 µL reaction was 2 µL 5 × Ion AmpliSeq HiFi mix, 5 µL primer pool, 10 ng (1–3 µL) DNA, and nuclease-free water (to 10 µL). The PCR profile was as follows: activation of the enzyme at 99°C for 2 min; 15 cycles of denaturation at 99°C for 15 s; and annealing and extension at 60°C for 8 min. Amplifications of the first and second pools of the Pan-Bacterial Research Panel were performed in separate reactions, which were then combined for further library preparation. Library preparation was performed according to the manufacturer’s protocol, and during these steps, the adapters containing the barcodes from the Ion Xpress Adapters barcode kits were also ligated to the amplicons. The libraries were purified with magnetic beads (Agencourt AMPure XP; Beckman Coulter, Brea, CA, USA) according to the manufacturer’s protocol. All libraries were quality checked and quantified using the DNA High Sensitivity assay on the LabChip GX capillary gel electrophoresis instrument (PerkinElmer, Inc., Hopkinton, MA, USA). Emulsion PCR for clonal amplification of the libraries on ion spheres, enrichment for spheres containing library, and loading of the chip were performed using the Ion Chef System (Thermo Fisher Scientific). For ion semiconductor sequencing, the Ion 530 chip and the Ion S5 system were used (Thermo Fisher Scientific).

#### Data analysis

The raw reads obtained during sequencing were analyzed using the Torrent Suite software PanBacterialAnalysis plug-in, which was specially developed for the analysis of reads from the Pan-Bacterial Research Panel. Reads with a length under 70 bp were filtered out and labeled as “invalid” in the output. Reads were classified by mapping against reference sequences within the plug-in (Greengenes public database for 16S rRNA sequences). Reads with local alignment scores over 35 were used and counted in the further analysis. Reads that were not aligned to any sequence in the database were reported as “unmapped.” Read counts for AMR genes and species-specific amplicons and 16S families were normalized separately. The normalized read counts for AMR genes and species-specific amplicons are reported as the ratio between the sum of the read counts of all amplicons for the species or gene of interest and the total read counts in that sample. The normalized 16S rRNA read count is reported as the ratio between the read counts of the family and the total reads mapped to the 16S rRNA reference in that sample. Only the AMR genes and the species-specific amplicons with normalized read counts over 0.1 and the 16S families with normalized read counts over 1.0 are reported as present in the samples. For 16S rRNA metagenomics, the results are reported as bacterial OTUs detected in the samples. For species-specific amplicons and for AMR genes, results are reported as the number of reads belonging to each amplicon. After automated data analysis, a threshold of 10 reads was applied for AMR genes and species-specific amplicons. Only AMR genes and bacterial species that reached this threshold were used for further analysis.

### Statistical data analysis

Kruskal-Wallis tests were performed to determine whether there was a statistically significant difference in DNA yield, genomic quality score, alpha-diversity and number of AMR genes due to the different DNA extraction methods, extraction days, and spiked sputum samples. The Benjamini-Hochberg method was used to control the false discovery rate. The beta-diversity of the microbiomes was analyzed using principal coordinate analysis (PCoA) based on Bray-Curtis dissimilarities. A permutational multivariate analysis of variance (PERMANOVA) was performed on Bray-Curtis dissimilarities to assess the effects of DNA extraction methods, extraction days, sputum samples and replicates on beta-diversity, and a pairwise PERMANOVA for the pairwise comparisons of the different groups. The same methods used to analyze and compare the microbiomes were also applied to the resistomes. All analyses were performed using the R programming language for statistical computing (23) and the Rstudio integrated development environment (24) in combination with the following packages: ggplot2 (25) and phyloseq (26) for visualization of the results, and vegan (27) and RVAideMemoire (28) for the diversity, dissimilarity, and multivariate analyses (29).

## RESULTS

### DNA yields and fragmentation with the different extraction methods

DNA was extracted from sputum samples in three technical replicates, each on 2 separate days, using one of three different DNA extraction methods and sequenced to determine the repeatability of the methods (Table S1). DNA yield and GQS differed significantly between the three extraction methods (*P*-value < 0.001; Kruskal-Wallis *t*-tests). The highest amount of DNA was extracted with the CTAB protocol (overall mean, 10.6 µg), followed by the GXT NA/Arrow kit (overall mean, 4.5 µg) and the QIAamp DNA mini kit (overall mean, 1.8 µg). Although the yield of the QIAamp DNA mini kit is the lowest, the quantity is sufficient for HTS analysis and is of high quality (highest GQS, overall mean of 2.4) and the highest repeatability (Fig. S3; Table S5). All three kits extracted DNA of sufficient quality and quantity to be used for library preparation for targeted HTS. Results were considered repeatable when the CV was below 25%. In terms of yield, all three extraction methods gave repeatable results. For DNA quality and fragmentation, defined as GQS using gel capillary electrophoresis, CVs were >25% in some cases for the CTAB protocol and the GXT NA/Arrow kits, indicating less consistent quality of extracted DNA (Table S5).

### Analysis of targeted high-throughput sequencing and alignment of raw reads

**Fig 2:**
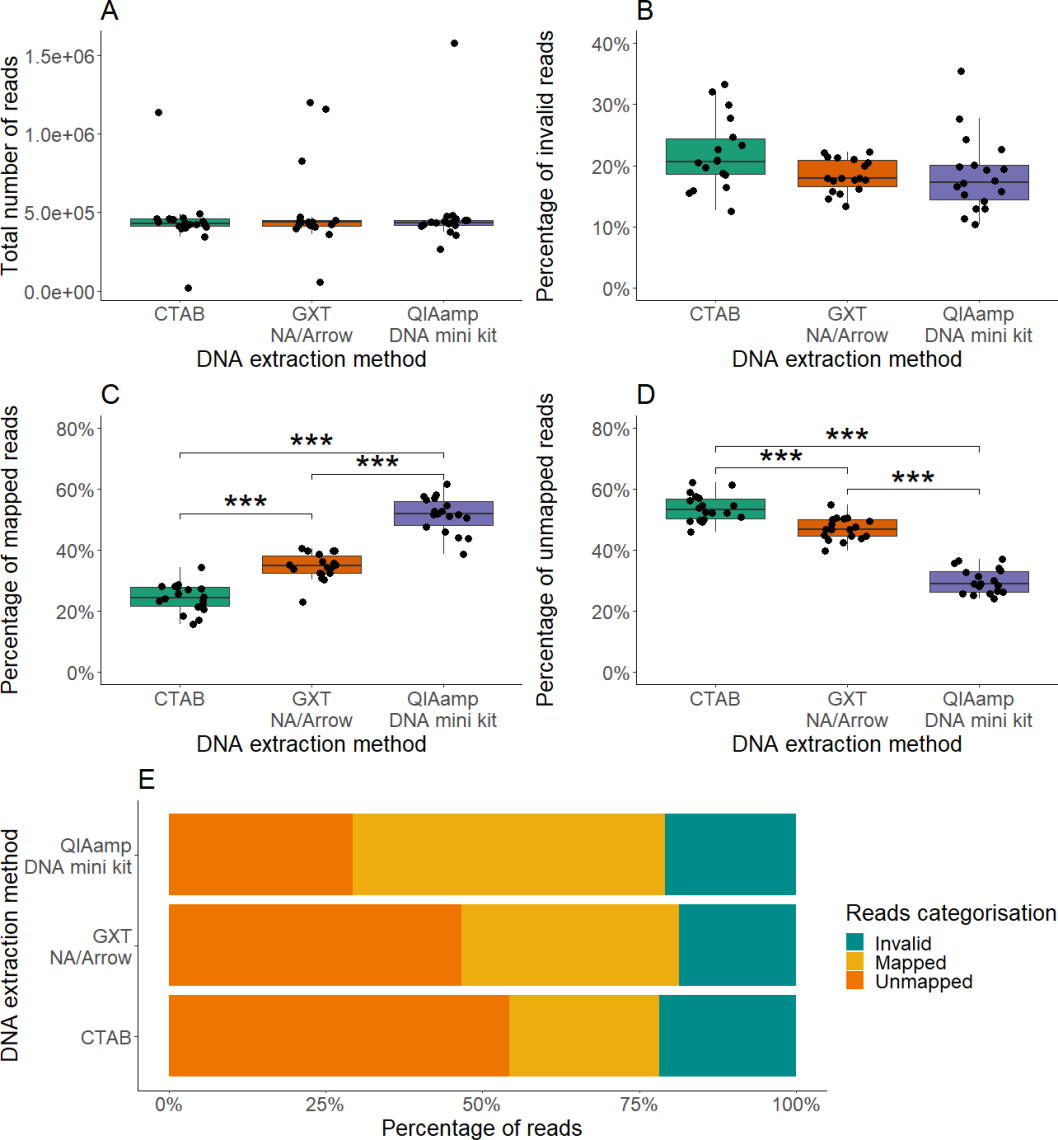
Results of data analysis after targeted HTS. Reads obtained for the high-throughput sequencing are colored according to the DNA extraction methods. (A–D) Boxplots showing medians and quartiles for the total number of reads (A), invalid reads (i.e., too short; not included in the analysis) (B), reads successfully mapped to reference sequences (C), and valid reads that did not map to any reference sequence (D). (E) Bar plot representations of the invalid mapped and unmapped reads for each extraction method (as indicated). ***, *P*-value < 0.001 (Kruskal-Wallis *t*-test).

The total number of reads obtained with the targeted was 2.60 × 10^7^, and the average number of reads per library was 4.81 × 10^5^ (CV, 52.1%). Both the differences in the total number of reads and the differences in the proportions of invalid reads between the DNA extraction methods were not significant (Fig. 2A and B; Table S6). There were significant differences in the proportions of mapped and unmapped reads between the three DNA extraction methods (*P*-values < 0.001, Kruskal-Wallis *t*-tests). In the samples extracted with the QIAamp DNA mini kit, the proportion of mapped reads was the highest (overall mean 52%) and the proportion of unmapped reads the lowest (30%). On the other hand, the CTAB protocol had the lowest proportion of mapped reads (overall mean 24%) and the highest proportion of unmapped reads (overall mean 54%) (Fig. 2C through E). Although samples extracted with the QIAamp DNA mini kit had the highest average percentage of mapped reads and the highest GQS, there was no significant correlation between the percentage of mapped reads and GQS in samples extracted with the same method.

#### Controls

Negative controls used for DNA extraction and library preparation were negative throughout the process. Amplifiable DNA was successfully extracted from all sputum samples as determined by DNA measurements (yield and quality), dPCR, and targeted HTS (specific detection of spiked bacteria). While dPCR pointed to no contaminations, HTS detected *A. baumannii* DNA in a CTAB negative control. This indicates that the contamination occurred during library preparation. The complex sputum background was followed throughout the process as a positive control. In addition, HTS sequencing of bacteria used for spiking identified species-specific and AMR amplicons (Table S7), matching genomic information (GenBank assembly accessions: GCA_009759685.1, JOOW00000000.1, GCA_001045685.1 Table S8).

#### Analytical sensitivity of targeted HTS for detection of bacteria in sputum samples

The sputum samples were spiked with relatively low bacterial concentrations, which are typical for the early phase of infection and persistent infections. All spiked sputum samples tested positive for the presence of spiked bacteria using dPCR (Tables S9 to S11). Targeted HTS and species-specific amplicons were used to detect spiked bacteria in sputum samples. As expected, reads belonging to spiked bacteria represent only a small fraction of the microbiome (<4%). We were able to detect *K. pneumoniae* and *A. baumannii* at high concentrations (5.4 × 10^5^ and 1.2 × 10^6^ cell/mL, respectively) with each of the DNA extraction methods, regardless of the days and technical replicates (Table 1). At the lowest concentrations, which are below the limit of detection for targeted HTS, we did not detect neither *A. baumannii* nor *K. pneumoniae* in any sample. The detection of *A. baumannii* and *K. pneumoniae* was inconsistent at intermediate concentrations (Table 1). In contrast to the other two spiked bacteria, we could not detect *P. aeruginosa* repeatedly*,* but only in a few samples (Table 1).

**TABLE 1:** Analytical sensitivity of targeted HTS detection of bacteria added to sputums^*a*^

Bacteria	Concentration of bacteria (cp/mL)	DNA extraction method					
		CTAB		GXT NA/Arrow		QIAamp DNA mini kit	
		Number	%	Number	%	Number	%
*Acinetobacter baumannii*	1.20 × 10^6^	6/6	100	6/6	100	6/6	100
	1.30 × 10^5^	0/6	0	4/6	67	6/6	100
	1.30 × 10^4^	0/6	0	0/6	0	0/6	0
*Klebsiella pneumoniae*	5.40 × 10^5^	5/6	83	6/6	100	6/6	100
	5.30 × 10^4^	0/6	0	1/6	17	2/6	33
	5.70 × 10^3^	0/6	0	0/6	0	0/6	0
*Pseudomonas aeruginosa*	1.20 × 10^6^	2/6	33	0/6	0	1/6	17
	1.30 × 10^5^	1/6	17	0/6	0	0/6	0
	1.20 × 10^4^	0/6	0	0/6	0	0/6	0

^*a*^ Proportion of samples (*n* = 6) in which the spiked bacteria at the different concentrations (low, medium, high) in the sputum samples were detected, according to the DNA extraction methods.

### Analytical sensitivity of targeted HTS for detection of AMR genes from spiked bacteria in sputum samples

Since spiked bacteria only make up a small part of the microbiome, the AMR genes belonging to these bacteria also only make up a small part of the resistome (<2%). All five AMR genes that were present in spiked bacteria were also detected in spiked sputum samples with high concentrations of spiked bacteria to which AMR genes belong. All genes were detected in samples of all extraction methods except for *macB* gene in samples extracted with CTAB. The percentages of detection for AMR genes ranged from 17 to 100% for the samples with high percentage of spiked bacteria. As expected, no AMR gene was detected in samples with low concentrations of spiked bacteria (Table 2). This indicates that these AMR genes are not part of the sputum microbiome and actually belong to the spiked bacteria. Compared to the detection of spiked bacteria with species-specific amplicons, some discrepancies were found. AMR genes were detected in a lower proportion of samples than *A. baumannii* and *K. pneumoniae*. In contrast, AMR genes from *P. aeruginosa* were detected in a much higher proportion of samples than the bacterium itself. This indicates that the overall sensitivity is related to the amplification efficiency of the different amplicons in a complex matrix. Similar to the detection of spiked bacteria, day of extraction and DNA extraction method did not show any significant effects on the detection of AMR genes from spiked bacteria.

**TABLE 2:** Analytical sensitivity of targeted HTS for detection of AMR genes^*a*^

Bacteria	AMR gene	Concentration of bacteria (cp/mL)	DNA extraction method					
			CTAB		GXT NA/Arrow		QIAamp DNA mini kit	
			Number	%	Number	%	Number	%
*Acinetobacter baumannii*	*eptA*	1.20 × 10^6^	6/6	100	5/6	83	6/6	100
		1.30 × 10^5^	0/6	0	0/6	0	0/6	0
		1.30 × 10^4^	0/6	0	0/6	0	0/6	0
	*uppP*	1.20 × 10^6^	4/6	67	5/6	83	4/6	67
		1.30 × 10^5^	0/6	0	0/6	0	0/6	0
		1.30 × 10^4^	0/6	0	0/6	0	0/6	0
*Klebsiella pneumoniae*	*macB*	5.40 × 10^5^	0/6	0	1/6	17	1/6	17
		5.30 × 10^4^	0/6	0	0/6	0	0/6	0
		5.70 × 10^3^	0/6	0	0/6	0	0/6	0
*Pseudomonas aeruginosa*	*aph(3') iib*	1.20 × 10^6^	5/6	83	2/6	33	3/6	50
		1.30 × 10^5^	0/6	0	0/6	0	0/6	0
		1.20 × 10^4^	0/6	0	0/6	0	0/6	0
	*catB7*	1.20 × 10^6^	5/6	83	2/6	33	2/6	33
		1.30 × 10^5^	1/6	17	0/6	0	0/6	0
		1.20 × 10^4^	0/6	0	0/6	0	0/6	0

^*a*^ Detection rates for the given AMR genes according to the concentrations of the bacteria and the DNA extraction methods.

### Evaluation of microbiome with 16S rRNA metagenomics

**Fig 3:**
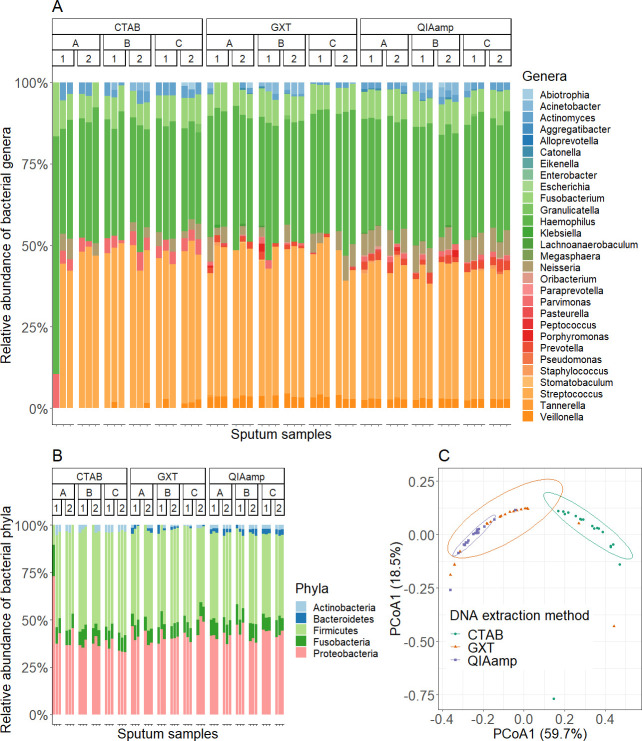
Comparison of microbiome richness and diversity from samples extracted with different DNA extraction methods. (A) Relative abundancies of the bacterial genera for the sputum samples according to the DNA extraction methods (CTAB, GXT, and QIAamp), sputum samples (A, B, and C), and days of extraction (1 and 2). Principal coordinate analysis in two dimensions (B) and constrained analysis of principal coordinates (C), using Bray Curtis distances and clustering of the samples according to the DNA extraction methods.

**Fig 4:**
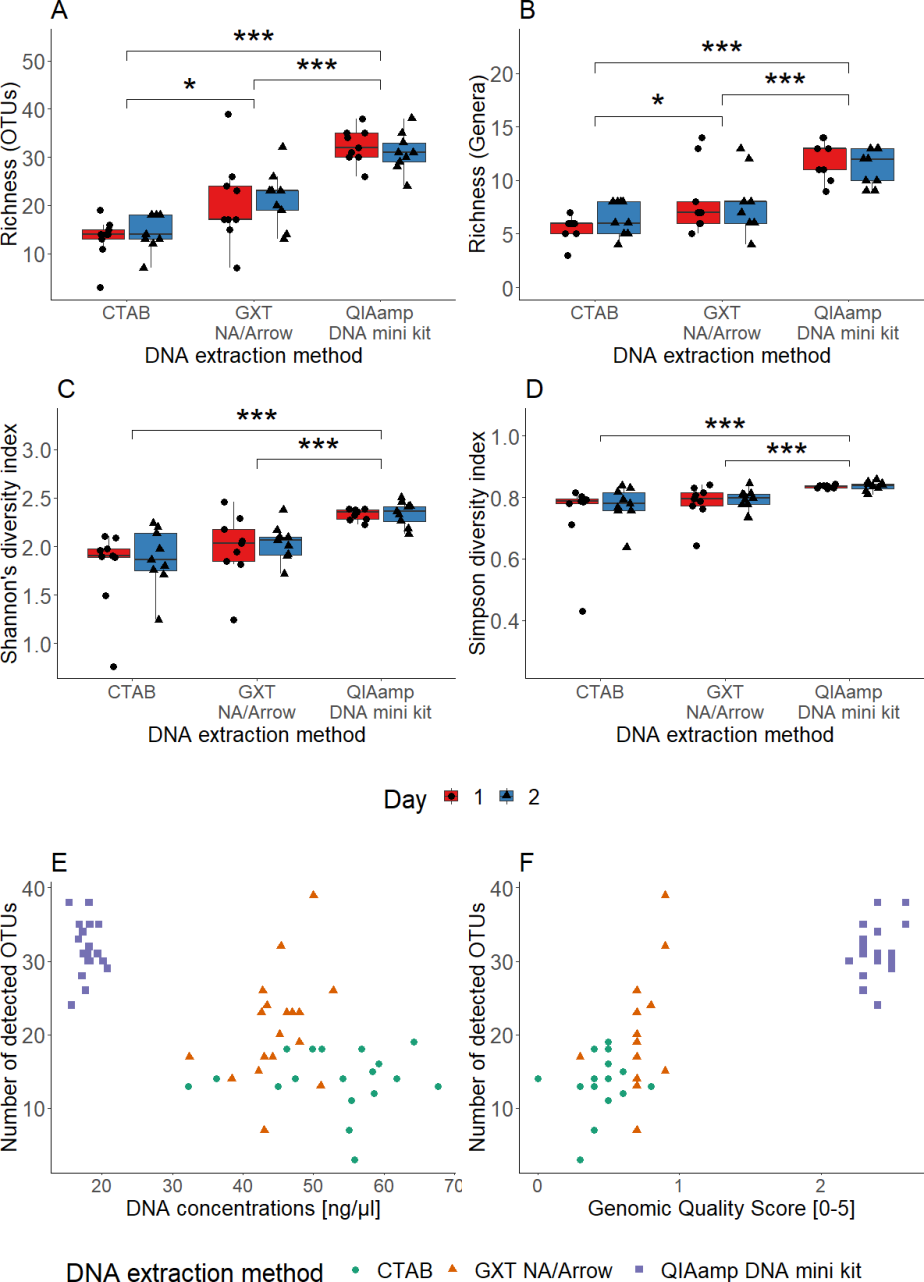
Alpha-diversity according to the DNA extraction methods, shown for the richness, as the number of OTUs (A), the number of genera (B), the Shannon diversity index (C), and the Simpson index (D). Effects on the OTUs detected in terms of DNA concentration (E) and genomic quality score (F) for the different samples. *, *P*-value < 0.05; ***, *P*-value < 0.001 (Kruskal-Wallis *t*-test).

The microbiome of spiked sputum samples was determined using targeted HTS of the 16S rRNA region. When looking at all sequenced samples together, we detected 80 different OTUs, 29 genera, and five phyla (Actinobacteria, Bacteroidetes, Firmicutes, Fusobacteria, and Proteobacteria) (Table S12; Fig. 3A and B). When looking at samples extracted by different method, a statistically significant effect on the microbiome was determined using Kruskal-Wallis *t*-test (Table S13). Beta-diversity of bacterial genera based on Bray-Curtis dissimilarities and two-dimensional PCoA were used for visualization. Samples were clustered by DNA extraction method, and there was some overlap between samples extracted with the QIAamp DNA mini kit and GXT NA/Arrow (Fig. 3). The DNA extraction method had a significant effect on the microbiome, explaining 63.2% of the total variance therein as calculated with PERMANOVA (Table S14). Richness (number of OTUs and genera) and diversity (Shannon’s and Simpson’s indices) were calculated for each sample. Samples extracted with the QIAamp DNA mini kit had significantly the highest richness and diversity (*P*-value < 0.001; Kruskal-Wallis *t*-tests, Table S15) and repeatability. In contrast, samples extracted using the CTAB protocol had the lowest richness and diversity (Fig. 4). No significant difference in richness and diversity was observed between extraction days, also indicating the repeatability of all three DNA extraction methods (Table S15). The method of DNA extraction also had a significant effect on the composition of the microbiome. The relative abundance of 10 bacterial genera differed significantly between the DNA extraction methods (Table S16). The proportion of Gram-positive bacteria was significantly higher in the samples extracted using the CTAB protocol (Tables S17 to S18).

### Evaluation of the resistome using targeted high-throughput screening of AMR genes

**Fig 5:**
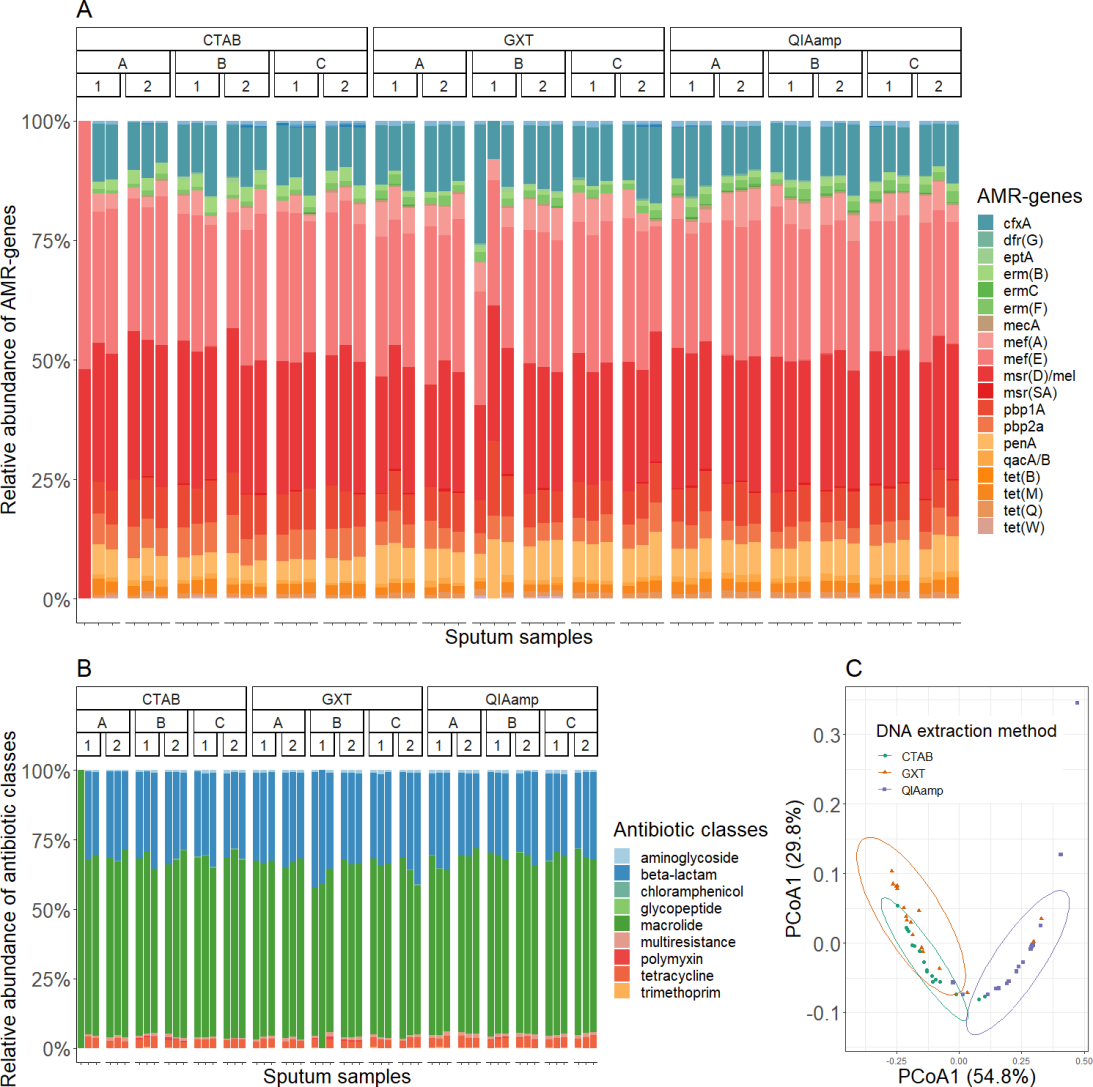
(A, B) Relative abundancies of the different AMR genes (A) and antimicrobial classes (B) for the sputum samples according to the DNA extraction methods (CTAB, GXT, and QIAamp), sputum samples (A, B, and C), and days of extraction (1 and 2). (C) Principal coordinate analysis using Bray-Curtis distances and clustering of the samples according to the DNA extraction methods. (D–F) The numbers of amplicons (D), AMR genes (E), and antimicrobial classes to which these genes possess resistance (F). The boxplots show median values and quartiles for each DNA extraction method and day of extraction. *, *P*-value < 0.05; **, *P*-value < 0.01; ***, *P*-value < 0.001 (Kruskal-Wallis *t*-test).

Together, we detected 50 amplicons and 27 AMR genes responsible for resistance to eight classes of antimicrobials (Fig. 5A and B; Table S19). AMR genes responsible for resistance to macrolides and beta-lactams were the most abundant, accounting for 64% and 31% of the resistome, respectively. Similar to the microbiome, the DNA extraction method had a statistically significant influence on the number of AMR genes and antimicrobial classes detected in the samples (*P*-value < 0.001; Kruskal-Wallis *t*-tests; Table S20). Again, beta-diversity of AMR genes based on Bray-Curtis dissimilarities and two-dimensional PCoA was used to visualize differences between samples. Samples extracted with the QIAamp DNA mini kit were grouped separately, while samples extracted with the CTAB protocol and GXT NA/Arrow overlapped (Fig. 5). The DNA extraction method had a significant effect on resistome, accounting for 54.2% of the total variance within samples as calculated with PERMANOVA (Table S21). Resistome diversity was analyzed by examining the number of AMR genes present in the samples and the corresponding antimicrobial classes. Samples extracted with the QIAamp DNA mini kit had significantly the highest number of AMR genes (overall mean 20.3) and corresponding antimicrobial classes (overall mean 6.7) as well as the most repeatable results. In contrast, the samples extracted with the CTAB protocol had the lowest number of AMR genes (overall mean 15.0) and the samples extracted with GXT NA/Arrow had the lowest number of antimicrobial classes and the lowest repeatability of the resistome (Fig. S4; Table S22). The DNA extraction method also influenced the composition of the resistome, with 26 AMR genes showing significantly different relative abundances (Table S23). The results described above clearly show that the DNA extraction method plays an important role in the determination of the resistome.

## DISCUSSION

In this study, the impact of different DNA extraction method on the results of targeted HTS for the microbiome and the resistome was critically assessed in a complex sputum matrix. This work addresses a gap in current research, as many previous studies have focused on mock communities rather than clinically relevant samples. By incorporating sputum, a highly heterogeneous matrix, findings of this study have direct applicability to diagnostic settings, enhancing the relevance and utility of results (14, 17, 30). Such clinical mock samples are also better suited to evaluate the repeatability and consistency of DNA extraction methods. Although there are many studies investigating the effects of DNA extraction methods on the microbiome, only a few of them focus on sputum. Due to the unique characteristics of each matrix, it is difficult to transfer the results of studies conducted with different matrices which is especially true for sputum. DNA extraction protocols and sample processing prior to DNA extraction have been shown in previous studies to have a significant impact on 16S rRNA metagenomics results (15, 20). For instance, mechanical lysis by bead beating and enzymatic lysis with lysozyme can significantly increase the percentage of Gram-positive bacteria that may otherwise be underrepresented (19, 31, 32). For sputum samples, the use of liquefying agents to homogenize the sputum samples is an important step in the treatment of samples prior to DNA extraction (16, 19).

In this study, three distinct DNA extraction methods were evaluated, involving different principles for DNA extraction. Each method demonstrated unique strengths and weaknesses. The CTAB protocol had the highest DNA yield but also the highest variability and fragmentation. The GXT NA/Arrow kit is commercially available and offers automated extraction based on binding of DNA to magnetic beads. Although it is automated, it did not show the highest repeatability. The QIAamp DNA mini kit is commercially available DNA extraction kit that is based on the binding of DNA to silica membranes using spin columns for extraction. It provided the most repeatable results with the least fragmented DNA, despite lower overall yield.

Targeted HTS with species-specific amplicons achieved detection limits as low as 10^4^ cells/mL for spiked bacteria, with variations in detection sensitivity among different species. *A. baumannii* was detected in the highest proportion of samples, *P. aeruginosa* in the lowest. The lower analytical sensitivity for *P. aeruginosa* than expected could be related to the lower efficiency of target amplification or the lower number of species-specific amplicons in the Pan-Bacterial Panel. Since similar differences in the detection rates of the different bacteria were found for both dPCR and targeted HTS, the lower efficiency of DNA extractions for the different bacterial species could also be the reason. The overall sensitivity is lower compared to dPCR, but with targeted HTS, a larger number of different bacterial species can be detected simultaneously.

The microbiome determination using targeted HTS of the 16S rRNA region provided repeatable results. The DNA extraction method showed a significant effect on the alpha-diversity parameters. Importantly, the QIAamp DNA mini kit consistently yielded the highest alpha-diversity and richness, suggesting it is the most suitable for comprehensive microbiome analysis. Both the CTAB protocol and the GXT NA/Arrow kit yielded a significantly lower number of detected OTUs and diversity in the samples. The presence or absence of low abundance species is one of the main reasons for the differences between the extraction methods. This could be due to the different dilution of the DNA prior to library preparation. Due to the higher concentration, the DNA extracted with the CTAB protocol had to be diluted significantly more, which could lead to a lower detection of low abundance species and consequently a lower diversity. The samples extracted with the CTAB protocol showed a higher proportion of Gram-positive bacteria which could be due to the enzymatic digestion with lysozyme (14, 16, 31). These insights are crucial for studies aiming to capture the full breadth of microbial diversity and resistome composition in clinical samples.

Similar trends were observed in the determination of the resistome, where the samples extracted with the QIAamp DNA mini kit showed a higher average number of detected AMR genes than with the CTAB protocol and the GXT NA/Arrow kit. However, the differences between the DNA extraction methods were less pronounced for the resistome than for the microbiome. This suggests that while the QIAamp DNA mini kit is particularly effective for microbiome studies, it is also a robust choice for resistome analysis. Even if the other two methods allow a more automated extraction, as with the GXT NA/Arrow kit, or are more cost-efficient, as with the CTAB protocol, these advantages seldom outweigh the higher repeatability and microbiome diversity of the results obtained with the QIAamp DNA mini kit. The number of samples in a typical study of the microbiome is generally not so high as to require automation, and the price of DNA extraction is only a small part of the overall price of targeted HTS. However, the automation could be integrated into DNA extraction methods based on the spin columns. Furthermore, the addition of enzymatic lysis with lysozyme prior to extraction to the QIAamp DNA mini kit protocol could enhance the detection of Gram-positive bacteria, improving the overall yield and providing results that even more accurately reflect the actual composition of the microbiome and resistome. Previous studies using mock communities with known abundance of different bacterial taxa have shown that different DNA extraction methods resulted in different microbiome compositions. However, no DNA extraction method gave results that perfectly reflected the actual composition, so we must be cautious when interpreting the differences between DNA extraction methods (31, 33).

Sequencing of the pure bacterial suspensions revealed that all three species used for spiking contained AMR genes which were also detected in the spiked sputum samples. The detection rates of these AMR genes increased with the concentrations of the spiked bacteria to which the AMR genes belong; this indicates that these AMR genes indeed originate from the spiked bacteria. However, at this stage, it cannot be completely ruled out that they belong to other bacteria in the sputum samples. It must also be emphasized that the AMR genes detected for the spiked bacteria and the sputum samples are not necessarily all genes associated with AMR in the bacteria and sputum, but only those that can be detected using the Pan-Bacterial Research Panel. An advantage of this panel is that it is based on genes associated with AMR rather than single nucleotide polymorphisms, which are less reliable for predicting AMR. The use of DNA extraction blank controls and no-template controls is necessary to identify potential contaminants that may occur during sample handling, DNA extraction, and library preparation. These controls are essential for accurate data interpretation and for minimizing the risk of false positives in microbiome studies (34).

While individual steps of DNA extraction (e.g., enzymatic lysis, use of solubilizing agents, bead beating, etc.) were not examined separately, comprehensive evaluation of the overall effects of three widely used DNA extraction methods enhances the transferability of results. In contrast to studies using mock communities, the actual composition of the microbiome is not known. Therefore, it cannot be determined how similar results of this study are to the actual composition of the microbiome. However, the sputum samples used here are much more representative of actual clinical samples.

In conclusion, the results of this study should serve as a guide for future research into the bacterial microbiome and resistome using targeted HTS, with particular emphasis on the importance of appropriate DNA extraction method. It is also shown that targeted HTS can provide repeatable results when the appropriate DNA extraction method is used, which allows for a more rationalized study design, avoiding potential errors and unsatisfactory results that may lead to prolonged studies and higher costs. By adopting the principles of metrology and diagnostics to targeted HTS, researchers can achieve better repeatability and comparability of results between laboratories, enhancing confidence in their findings and contributing to the advancement of microbiome and resistome research.

## Data Availability

Data used in this study are publicly available at https://zenodo.org/records/11619401.

## SUPPLEMENTAL MATERIAL

Supplemental material msystems.00735-24-s0001.docx Supplemental figures and tables.

ASM does not own the copyrights to Supplemental Material that may be linked to, or accessed through, an article. The authors have granted ASM a non-exclusive, world-wide license to publish the Supplemental Material files. Please contact the corresponding author directly for reuse.
