# Supplementary material for: Metrological evaluation of DNA extraction method effects on the bacterial microbiome and resistome in sputum
Source: mSystems. 2024 Aug 16;9(9):e00735-24. doi: 10.1128/msystems.00735-24 (PMC11406916; doi:10.1128/msystems.00735-24)

Supplemental materials

# **Tables**

Table S1

Properties of the three DNA extraction methods used in the study.

| **Type** | **Method name** | **Extraction principle** | **Separation method** | **Extraction format** | **Cell lysis** |
| --- | --- | --- | --- | --- | --- |
| In-house | CTAB protocol | Solution based | Selective precipitation | Manual extraction | Lysozyme, lysis buffer (CTAB, sodium dodecyl sulphate, salt), proteinase K |
| Commercial kits | GXT NA/Arrow kit | Solid phase | Binding to magnetic beads | Automated extraction | Lysis buffer (guanidine thiocyanate, detergent, salt), proteinase K |
|  | QIAamp DNA mini kit | Solid phase | Binding to silica membrane | Spin-column format | Lysis buffer (sodium dodecyl sulphate, guanidine hydrochloride), proteinase K |

Table S2

Concentrations of the spiked bacteria in samples A, B and C according to the turbidimetry McFarland standards (McF) and as determined using dPCR of cells in suspension without extraction of DNA (Actual).

| **Method** | **Bacteria** | **Bacterial concentration in sputum sample (cp/mL)** | | | | | |
| --- | --- | --- | --- | --- | --- | --- | --- |
|  |  | **A** | | **B** | | **C** | |
|  |  | **Target** | **Measured** | **Target** | **Measured** | **Target** | **Measured** |
| **dPCR** | *A. baumannii* | Medium | 1.30 ×10^5^ | High | 1.20 ×10^6^ | Low | 1.30 ×10^4^ |
|  | *K. pneumoniae* | High | 5.40 ×10^5^ | Low | 5.70 ×10^3^ | Medium | 5.30 ×10^4^ |
|  | *P. aeruginosa* | Low | 1.20 ×10^4^ | Medium | 1.30 ×10^5^ | High | 1.20 ×10^6^ |
| **McF** | *A. baumannii* | Medium | 5.5 ×10^4^ | High | 5.5 ×10^5^ | Low | 5.5 ×10^3^ |
|  | *K. pneumoniae* | High | 5.5 ×10^5^ | Low | 5.5 ×10^3^ | Medium | 5.5 ×10^4^ |
|  | *P. aeruginosa* | Low | 5.5 ×10^3^ | Medium | 5.5 ×10^4^ | High | 5.5 ×10^5^ |

Table S3

Primers and probes used in the dPCR quantification. ^1^All assays were developed in-house, ^2^Forward primer, ^3^Reverse primer, ^4^Probe – all probes were FAM/MGB

| **Target^1^** | **Oligo** | ***Sequence*** | ***Amplicon length*** | ***Final concentration (nM)*** |
| --- | --- | --- | --- | --- |
| ***A. baumannii*** | F^2^ | GCTCGTGATTCGACTCAAATCA |  | 900 |
|  | R^3^ | GCAAACGAATAATTTAACCATGCTT | 68 bp | 900 |
|  | P^4^ | CTGATTAGCCAAGTTGC |  | 300 |
| ***K. pneumoniae*** | F | CCCGCTGTGGTAATACCCTACT |  | 900 |
|  | R | GCTGCGTCAGGCAAATCTTC | 62 bp | 900 |
|  | P | CAGAGGGAACTGGC |  | 300 |
| ***P. aeruginosa*** | F | TCTGCCGCGGGTTCTTC |  | 900 |
|  | R | AGGATACCTACGCCCAGTTGCT | 64 bp | 900 |
|  | P | CTTCCAGCAGGGACAC |  | 300 |

Table S4

List of microorganisms which can be detected using species specific amplicons from first pool of Ion Ampliseq Pan-Bacterial Research Panel.

| Microorganism name |
| --- |
| *Acinetobacter baumannii* |
| *Candida albicans* |
| *Citrobacter freundii* |
| *Enterobacter cloacae* |
| *Enterococcus faecalis* |
| *Enterococcus faecium* |
| *Escherichia coli* |
| *Haemophilus influenzae* |
| *Klebsiella oxytoca* |
| *Klebsiella pneumoniae* |
| *Neisseria meningitidis* |
| *Proteus mirabilis* |
| *Pseudomonas aeruginosa* |
| *Serratia marcescens* |
| *Staphylococcus aureus* |
| *Staphylococcus epidermidis* |
| *Staphylococcus haemolyticus* |
| *Streptococcus pneumoniae* |
| *Streptococcus pyogenes* |
| *Streptococcus salivarius* |

Table S5

Table showing the DNA yield (as µg of extracted DNA), and fragmentation of DNA (as genomic quality score [GQS]) with different DNA extraction methods CTAB, GXT NA/Arrow and QIAamp DNA mini kit. Values are given for two separate days of extraction and for both days combined.

| **Parameter** | **Day** | **Data obtained according to DNA extraction method** | | | | | | | | |
| --- | --- | --- | --- | --- | --- | --- | --- | --- | --- | --- |
|  |  | **CTAB** | | | **GXT NA/Arrow** | | | **QIAamp DNA mini kit** | | |
|  |  | **Range** | **Mean** | **CV (%)** | **Range** | **Mean** | **CV (%)** | **Range** | **Mean** | **CV (%)** |
| DNA yield (µg) | 1 | 9.0-12.8 | 11.1 | 11 | 3.2-5.0 | 4.3 | 11 | 1.5-2.0 | 1.8 | 7.2 |
|  | 2 | 6.4-13.5 | 10.1 | 22 | 3.8-5.3 | 4.7 | 9.2 | 1.6-2.1 | 1.8 | 8.9 |
|  | Both | 6.4-13.5 | 10.6 | 17 | 3.2-5.3 | 4.5 | 11 | 1.5-2.1 | 1.8 | 7.9 |
| Genomic Quality Score (GQS) | 1 | 0.3-0.6 | 0.5 | 19 | 0.3-0.9 | 0.7 | 25 | 2.2-2.6 | 2.3 | 4.8 |
|  | 2 | 0.3-0.8 | 0.5 | 32 | 0.7-0.9 | 0.7 | 9.2 | 2.3-2.6 | 2.4 | 4.1 |
|  | Both | 0.3-0.8 | 0.5 | 25 | 0.3-0.9 | 0.7 | 18 | 2.2-2.6 | 2.4 | 5.0 |

Table S6

Tabel shows the percentages of mapped, unmapped and invalid reads with different DNA extraction methods CTAB, GXT NA/Arrow and QIAamp DNA mini kit. Values are given for two separate days of extraction and for both days combined.

| **Parameter** | **Day** | **Data obtained according to DNA extraction method** | | | | | | | | |
| --- | --- | --- | --- | --- | --- | --- | --- | --- | --- | --- |
|  |  | **CTAB** | | | **GXT** | | | **QIAamp DNA mini kit** | | |
|  |  | **Range** | **Mean** | **CV (%)** | **Range** | **Mean** | **CV (%)** | **Range** | **Mean** | **CV (%)** |
| Mapped reads (%) | 1 | 16-28 | 22 | 19 | 32-40 | 35 | 7.2 | 39-58 | 48 | 13 |
|  | 2 | 18-34 | 27 | 16 | 23-40 | 34 | 17 | 51-62 | 55 | 6.6 |
|  | Both | 16-34 | 24 | 19 | 23-40 | 35 | 12 | 39-62 | 52 | 11 |
| Unmapped reads (%) | 1 | 46-62 | 54 | 9.2 | 42-51 | 47 | 6.6 | 26-37 | 31 | 11 |
|  | 2 | 49-62 | 53 | 7.6 | 40-55 | 47 | 9.1 | 24-37 | 29 | 17 |
|  | Both | 46-62 | 54 | 8.3 | 40-55 | 47 | 7.7 | 24-37 | 30 | 14 |
| Invalid reads (%) | 1 | 16-33 | 24 | 12 | 13-21 | 17 | 15 | 13-36 | 21 | 23 |
|  | 2 | 13-28 | 20 | 8.3 | 16-22 | 19 | 5.4 | 10-24 | 16 | 15 |
|  | Both | 13-33 | 22 | 27 | 13-22 | 18 | 15 | 10-36 | 18 | 34 |

Table S7

Antimicrobial resistance (AMR) genes detected in each of the bacterial species with targeted HTS using Ion AmpliSeq Pan-Bacterial Research Panel. Each AMR gene was detected with two amplicons.

| Bacteria | AMR gene | Encoded protein | Antimicrobial resistance |
| --- | --- | --- | --- |
| *Acinetobacter baumannii* | *eptA* | Lipid A phosphoethanolamine transferase | Polymixyns |
|  | *uppP* | Undecaprenyl-diphosphate phosphatase | Bacitracin |
| *Klebsiella pneumoniae* | *macB* | Macrolide ABC transporter ATP-binding protein/permease MacB | Macrolides |
| *Pseudomonas aeruginosa* | *aph(3') iib* | Aminoglycoside O-phosphotransferase APH(3')-IIb | Aminoglycosides |
|  | *catB7* | Type B-4 chloramphenicol O-acetyltransferase CatB7 | Chloramphenicol |

Table S8

Table showing GenBank accession numbers of the genome assemblies of bacterial strains used for the preparation of spiked sputum samples.

| Bacteria | Strain | GenBank assembly accession |
| --- | --- | --- |
| *Acinetobacter baumannii* | DSMZ 30007 | GCA_009759685.1 |
| *Klebsiella pneumoniae* | DSMZ 30104 | GCA_000742135.1 |
| *Pseudomonas aeruginosa* | DSMZ 50071 | GCA_001045685.1 |

Table S9

Results of dPCR for sample A, showing mean copy number per reaction and ml of sample with CV% per sample.

| **Extraction** | **Day** | **Extraction parallel** | **Target bacteria** | **mean cp/rnx** | **cp/mL sample** | **CV%**  **sample** |
| --- | --- | --- | --- | --- | --- | --- |
| CTAB | 1 | 1 | *A. baumannii* | 479 | 119820 | 7.26 |
| CTAB | 1 | 2 | *A. baumannii* | 489 | 122197 | 7.74 |
| CTAB | 1 | 3 | *A. baumannii* | 440 | 110017 | 9.15 |
| CTAB | 2 | 1 | *A. baumannii* | 346 | 86412 | 5.83 |
| CTAB | 2 | 2 | *A. baumannii* | 444 | 111049 | 7.91 |
| CTAB | 2 | 3 | *A. baumannii* | 512 | 127943 | 8.67 |
| CTAB | 1 | 1 | *K. pneumoniae* | 2187 | 546699 | 11.41 |
| CTAB | 1 | 2 | *K. pneumoniae* | 2271 | 567687 | 6.70 |
| CTAB | 1 | 3 | *K. pneumoniae* | 2161 | 540136 | 1.99 |
| CTAB | 2 | 1 | *K. pneumoniae* | 1646 | 411438 | 5.61 |
| CTAB | 2 | 2 | *K. pneumoniae* | 2088 | 521974 | 3.93 |
| CTAB | 2 | 3 | *K. pneumoniae* | 2447 | 611723 | 2.88 |
| CTAB | 1 | 1 | *P. aeruginosa* | 247 | 61825 | 16.52 |
| CTAB | 1 | 2 | *P. aeruginosa* | 427 | 106848 | 3.41 |
| CTAB | 1 | 3 | *P. aeruginosa* | 1065 | 266151 | 4.48 |
| CTAB | 2 | 1 | *P. aeruginosa* | 180 | 45026 | 10.32 |
| CTAB | 2 | 2 | *P. aeruginosa* | 1163 | 290862 | 1.93 |
| CTAB | 2 | 3 | *P. aeruginosa* | 353 | 88270 | 10.74 |
| GXT_NA | 1 | 1 | *A. baumannii* | 1345 | 168144 | 14.26 |
| GXT_NA | 1 | 2 | *A. baumannii* | 1549 | 193635 | 4.39 |
| GXT_NA | 1 | 3 | *A. baumannii* | 1439 | 179836 | 1.78 |
| GXT_NA | 2 | 1 | *A. baumannii* | 1313 | 164179 | 6.16 |
| GXT_NA | 2 | 2 | *A. baumannii* | 1425 | 178102 | 1.22 |
| GXT_NA | 2 | 3 | *A. baumannii* | 1386 | 173280 | 3.42 |
| GXT_NA | 1 | 1 | *K. pneumoniae* | 7357 | 919646 | 0.80 |
| GXT_NA | 1 | 2 | *K. pneumoniae* | 7700 | 962446 | 3.30 |
| GXT_NA | 1 | 3 | *K. pneumoniae* | 7376 | 921950 | 3.31 |
| GXT_NA | 2 | 1 | *K. pneumoniae* | 7056 | 881976 | 4.80 |
| GXT_NA | 2 | 2 | *K. pneumoniae* | 7538 | 942254 | 3.12 |
| GXT_NA | 2 | 3 | *K. pneumoniae* | 7289 | 911134 | 1.85 |
| GXT_NA | 1 | 1 | *P. aeruginosa* | 157 | 19626 | 20.31 |
| GXT_NA | 1 | 2 | *P. aeruginosa* | 183 | 22868 | 11.19 |
| GXT_NA | 1 | 3 | *P. aeruginosa* | 154 | 19234 | 23.19 |
| GXT_NA | 2 | 1 | *P. aeruginosa* | 166 | 20762 | 18.05 |
| GXT_NA | 2 | 2 | *P. aeruginosa* | 211 | 26416 | 10.11 |
| GXT_NA | 2 | 3 | *P. aeruginosa* | 183 | 22910 | 10.99 |
| QIAamp DNAminikit | 1 | 1 | *A. baumannii* | 243 | 30428 | 9.54 |
| QIAamp DNAminikit | 1 | 2 | *A. baumannii* | 269 | 33568 | 20.75 |
| QIAamp DNAminikit | 1 | 3 | *A. baumannii* | 283 | 35331 | 7.86 |

Continuation of Table S9

| **Extraction** | **Day** | **Extraction parallel** | **Target bacteria** | **mean cp/rnx** | **cp/mL sample** | **CV%**  **sample** |
| --- | --- | --- | --- | --- | --- | --- |
| QIAamp DNAminikit | 2 | 1 | *A. baumannii* | 327 | 40926 | 18.61 |
| QIAamp DNAminikit | 2 | 2 | *A. baumannii* | 392 | 49017 | 9.75 |
| QIAamp DNAminikit | 2 | 3 | *A. baumannii* | 349 | 43665 | 17.48 |
| QIAamp DNAminikit | 1 | 1 | *K. pneumoniae* | 1680 | 209948 | 3.33 |
| QIAamp DNAminikit | 1 | 2 | *K. pneumoniae* | 1686 | 210704 | 5.38 |
| QIAamp DNAminikit | 1 | 3 | *K. pneumoniae* | 1761 | 220076 | 2.38 |
| QIAamp DNAminikit | 2 | 1 | *K. pneumoniae* | 1627 | 203413 | 2.69 |
| QIAamp DNAminikit | 2 | 2 | *K. pneumoniae* | 1776 | 222023 | 5.12 |
| QIAamp DNAminikit | 2 | 3 | *K. pneumoniae* | 1923 | 240387 | 4.06 |
| QIAamp DNAminikit | 1 | 1 | *P. aeruginosa* | 36 | 4460 | 17.80 |
| QIAamp DNAminikit | 1 | 2 | *P. aeruginosa* | 36 | 4467 | 46.94 |
| QIAamp DNAminikit | 1 | 3 | *P. aeruginosa* | 42 | 5266 | 4.01 |
| QIAamp DNAminikit | 2 | 1 | *P. aeruginosa* | 38 | 4791 | 23.27 |
| QIAamp DNAminikit | 2 | 2 | *P. aeruginosa* | 41 | 5115 | 14.31 |
| QIAamp DNAminikit | 2 | 3 | *P. aeruginosa* | 36 | 4554 | 10.58 |

Table S10

Results of dPCR for sample B, showing mean copy number per reaction and ml of sample with CV% per sample.

| **Extraction** | **Day** | **Extraction parallel** | **Target bacteria** | **mean cp/rnx** | **cp/mL sample** | **CV%**  **sample** |
| --- | --- | --- | --- | --- | --- | --- |
| CTAB | 1 | 1 | *A. baumannii* | 4887 | 1221790 | 7.11 |
| CTAB | 1 | 2 | *A. baumannii* | 4668 | 1166972 | 9.20 |
| CTAB | 1 | 3 | *A. baumannii* | 4884 | 1220921 | 1.86 |
| CTAB | 2 | 1 | *A. baumannii* | 4844 | 1211008 | 0.71 |
| CTAB | 2 | 2 | *A. baumannii* | 2175 | 543670 | 4.13 |
| CTAB | 2 | 3 | *A. baumannii* | 5025 | 1256179 | 1.31 |
| CTAB | 1 | 1 | *K. pneumoniae* | 17 | 4310 | 8.50 |
| CTAB | 1 | 2 | *K. pneumoniae* | 33 | 8297 | 29.90 |
| CTAB | 1 | 3 | *K. pneumoniae* | 20 | 4974 | 13.65 |
| CTAB | 2 | 1 | *K. pneumoniae* | 26 | 6386 | 37.24 |
| CTAB | 2 | 2 | *K. pneumoniae* | 10 | 3161 | 1.10 |
| CTAB | 2 | 3 | *K. pneumoniae* | 24 | 5938 | 20.01 |
| CTAB | 1 | 1 | *P. aeruginosa* | 3240 | 810111 | 1.17 |
| CTAB | 1 | 2 | *P. aeruginosa* | 1493 | 373169 | 2.83 |
| CTAB | 1 | 3 | *P. aeruginosa* | 1602 | 400552 | 3.11 |
| CTAB | 2 | 1 | *P. aeruginosa* | 1411 | 352693 | 2.52 |
| CTAB | 2 | 2 | *P. aeruginosa* | 1532 | 382970 | 6.02 |
| CTAB | 2 | 3 | *P. aeruginosa* | 2412 | 602877 | 4.46 |
| GXT_NA | 1 | 1 | *A. baumannii* | 14403 | 1800346 | 1.92 |
| GXT_NA | 1 | 2 | *A. baumannii* | 15588 | 1948512 | 3.01 |
| GXT_NA | 1 | 3 | *A. baumannii* | 14493 | 1811585 | 4.64 |
| GXT_NA | 2 | 1 | *A. baumannii* | 14949 | 1868677 | 3.59 |
| GXT_NA | 2 | 2 | *A. baumannii* | 14836 | 1854556 | 5.65 |
| GXT_NA | 2 | 3 | *A. baumannii* | 14194 | 1774262 | 2.38 |
| GXT_NA | 1 | 1 | *K. pneumoniae* | 67 | 8317 | 10.60 |
| GXT_NA | 1 | 2 | *K. pneumoniae* | 74 | 9212 | 23.62 |
| GXT_NA | 1 | 3 | *K. pneumoniae* | 71 | 8910 | 7.68 |
| GXT_NA | 2 | 1 | *K. pneumoniae* | 65 | 8105 | 20.17 |
| GXT_NA | 2 | 2 | *K. pneumoniae* | 76 | 9463 | 6.20 |
| GXT_NA | 2 | 3 | *K. pneumoniae* | 95 | 11878 | 11.19 |
| GXT_NA | 1 | 1 | *P. aeruginosa* | 1539 | 192411 | 6.61 |
| GXT_NA | 1 | 2 | *P. aeruginosa* | 1649 | 206176 | 3.37 |
| GXT_NA | 1 | 3 | *P. aeruginosa* | 1589 | 198651 | 5.26 |
| GXT_NA | 2 | 1 | *P. aeruginosa* | 1631 | 203864 | 5.26 |
| GXT_NA | 2 | 2 | *P. aeruginosa* | 1721 | 215088 | 4.83 |
| GXT_NA | 2 | 3 | *P. aeruginosa* | 1534 | 191792 | 5.04 |
| QIAamp DNAminikit | 1 | 1 | *A. baumannii* | 3225 | 403093 | 3.24 |
| QIAamp DNAminikit | 1 | 2 | *A. baumannii* | 3224 | 403033 | 2.30 |
| QIAamp DNAminikit | 1 | 3 | *A. baumannii* | 2991 | 373817 | 6.27 |

Continuation of Table S10

| **Extraction** | **Day** | **Extraction parallel** | **Target bacteria** | **mean cp/rnx** | **cp/mL sample** | **CV%**  **sample** |
| --- | --- | --- | --- | --- | --- | --- |
| QIAamp DNAminikit | 2 | 2 | *A. baumannii* | 4177 | 522156 | 3.96 |
| QIAamp DNAminikit | 2 | 3 | *A. baumannii* | 3365 | 420579 | 2.23 |
| QIAamp DNAminikit | 1 | 1 | *K. pneumoniae* | 24 | 3017 | 10.71 |
| QIAamp DNAminikit | 1 | 2 | *K. pneumoniae* | 14 | 1797 | 50.58 |
| QIAamp DNAminikit | 1 | 3 | *K. pneumoniae* | 15 | 1905 | 54.15 |
| QIAamp DNAminikit | 2 | 1 | *K. pneumoniae* | 19 | 2391 | 12.69 |
| QIAamp DNAminikit | 2 | 2 | *K. pneumoniae* | 23 | 2873 | 48.16 |
| QIAamp DNAminikit | 2 | 3 | *K. pneumoniae* | 14 | 1688 | 15.46 |
| QIAamp DNAminikit | 1 | 1 | *P. aeruginosa* | 326 | 40709 | 3.76 |
| QIAamp DNAminikit | 1 | 2 | *P. aeruginosa* | 364 | 45481 | 7.52 |
| QIAamp DNAminikit | 1 | 3 | *P. aeruginosa* | 298 | 37296 | 3.46 |
| QIAamp DNAminikit | 2 | 1 | *P. aeruginosa* | 340 | 42524 | 12.97 |
| QIAamp DNAminikit | 2 | 2 | *P. aeruginosa* | 334 | 41707 | 4.42 |
| QIAamp DNAminikit | 2 | 3 | *P. aeruginosa* | 262 | 32798 | 5.57 |

Table S11

Results of dPCR for sample C, showing mean copy number per reaction and ml of sample with CV% per sample.

| **Extraction** | **Day** | **Extraction parallel** | **Target bacteria** | **mean cp/rnx** | **cp/mL sample** | **CV%**  **sample** |
| --- | --- | --- | --- | --- | --- | --- |
| CTAB | 1 | 1 | *A. baumannii* | 69 | 17177 | 21.58 |
| CTAB | 1 | 2 | *A. baumannii* | 51 | 12707 | 23.51 |
| CTAB | 1 | 3 | *A. baumannii* | 40 | 9990 | 7.84 |
| CTAB | 2 | 1 | *A. baumannii* | 62 | 15458 | 7.96 |
| CTAB | 2 | 2 | *A. baumannii* | 58 | 14408 | 15.15 |
| CTAB | 2 | 3 | *A. baumannii* | 42 | 10581 | 16.79 |
| CTAB | 1 | 1 | *K. pneumoniae* | 192 | 47937 | 56.05 |
| CTAB | 1 | 2 | *K. pneumoniae* | 232 | 57922 | 1.87 |
| CTAB | 1 | 3 | *K. pneumoniae* | 153 | 38354 | 22.84 |
| CTAB | 2 | 1 | *K. pneumoniae* | 246 | 61575 | 4.40 |
| CTAB | 2 | 2 | *K. pneumoniae* | 233 | 58167 | 12.26 |
| CTAB | 2 | 3 | *K. pneumoniae* | 195 | 48811 | 13.38 |
| CTAB | 1 | 1 | *P. aeruginosa* | 7209 | 1802250 | 1.61 |
| CTAB | 1 | 2 | *P. aeruginosa* | 8044 | 2011103 | 4.96 |
| CTAB | 1 | 3 | *P. aeruginosa* | 5334 | 1333460 | 4.68 |
| CTAB | 2 | 1 | *P. aeruginosa* | 6449 | 1612354 | 5.34 |
| CTAB | 2 | 2 | *P. aeruginosa* | 7339 | 1834811 | 3.70 |
| CTAB | 2 | 3 | *P. aeruginosa* | 8378 | 2094533 | 1.15 |
| GXT_NA | 1 | 1 | *A. baumannii* | 185 | 23071 | 7.31 |
| GXT_NA | 1 | 2 | *A. baumannii* | 138 | 17285 | 10.91 |
| GXT_NA | 1 | 3 | *A. baumannii* | 149 | 18685 | 19.27 |
| GXT_NA | 2 | 1 | *A. baumannii* | 167 | 20908 | 7.30 |
| GXT_NA | 2 | 2 | *A. baumannii* | 147 | 18347 | 11.26 |
| GXT_NA | 2 | 3 | *A. baumannii* | 133 | 16674 | 7.88 |
| GXT_NA | 1 | 1 | *K. pneumoniae* | 760 | 95005 | 11.46 |
| GXT_NA | 1 | 2 | *K. pneumoniae* | 785 | 98121 | 8.28 |
| GXT_NA | 1 | 3 | *K. pneumoniae* | 741 | 92647 | 1.88 |
| GXT_NA | 2 | 1 | *K. pneumoniae* | 843 | 105389 | 7.85 |
| GXT_NA | 2 | 2 | *K. pneumoniae* | 753 | 94087 | 10.70 |
| GXT_NA | 2 | 3 | *K. pneumoniae* | 744 | 92971 | 6.26 |
| GXT_NA | 1 | 1 | *P. aeruginosa* | 16651 | 2081318 | 1.05 |
| GXT_NA | 1 | 2 | *P. aeruginosa* | 16986 | 2123268 | 2.31 |
| GXT_NA | 1 | 3 | *P. aeruginosa* | 16148 | 2018540 | 2.07 |
| GXT_NA | 2 | 1 | *P. aeruginosa* | 18716 | 2339460 | 3.00 |
| GXT_NA | 2 | 2 | *P. aeruginosa* | 16091 | 2011325 | 4.44 |
| GXT_NA | 2 | 3 | *P. aeruginosa* | 17030 | 2128742 | 7.54 |
| QIAamp DNAminikit | 1 | 1 | *A. baumannii* | 39 | 4921 | 30.82 |
| QIAamp DNAminikit | 1 | 2 | *A. baumannii* | 43 | 5358 | 9.31 |
| QIAamp DNAminikit | 1 | 3 | *A. baumannii* | 40 | 5039 | 17.43 |
| QIAamp DNAminikit | 2 | 1 | *A. baumannii* | 47 | 5849 | 45.60 |

Continuation of Table S11

| **Extraction** | **Day** | **Extraction parallel** | **Target bacteria** | **mean cp/rnx** | **cp/mL sample** | **CV%**  **sample** |
| --- | --- | --- | --- | --- | --- | --- |
| QIAamp DNAminikit | 2 | 2 | *A. baumannii* | 41 | 5067 | 6.07 |
| QIAamp DNAminikit | 2 | 3 | *A. baumannii* | 34 | 4272 | 26.83 |
| QIAamp DNAminikit | 1 | 1 | *K. pneumoniae* | 234 | 29221 | 9.49 |
| QIAamp DNAminikit | 1 | 2 | *K. pneumoniae* | 221 | 27578 | 20.04 |
| QIAamp DNAminikit | 1 | 3 | *K. pneumoniae* | 225 | 28153 | 14.39 |
| QIAamp DNAminikit | 2 | 1 | *K. pneumoniae* | 231 | 28827 | 12.67 |
| QIAamp DNAminikit | 2 | 2 | *K. pneumoniae* | 213 | 26567 | 9.24 |
| QIAamp DNAminikit | 2 | 3 | *K. pneumoniae* | 178 | 22274 | 6.72 |
| QIAamp DNAminikit | 1 | 1 | *P. aeruginosa* | 4284 | 535515 | 3.06 |
| QIAamp DNAminikit | 1 | 2 | *P. aeruginosa* | 4434 | 554250 | 4.66 |
| QIAamp DNAminikit | 1 | 3 | *P. aeruginosa* | 4553 | 569101 | 0.23 |
| QIAamp DNAminikit | 2 | 1 | *P. aeruginosa* | 4365 | 545633 | 2.79 |
| QIAamp DNAminikit | 2 | 2 | *P. aeruginosa* | 4379 | 547424 | 3.56 |
| QIAamp DNAminikit | 2 | 3 | *P. aeruginosa* | 3926 | 490705 | 1.40 |

Table S12

Alpha diversity parameters of microbiome of spiked sputum samples. Table shows minimal, maximal (range) and average (mean) number of Operational taxonomic units (OTUs), genera, Shannon indices and Simpson indices for three DNA extraction methods CTAB, GXT NA/Arrow and QIAamp DNA mini kit. Values are given for two separate days of extraction and for both days combined.

| **Parameter** | **Day** | **Data obtained according to DNA extraction method** | | | | | | | | |
| --- | --- | --- | --- | --- | --- | --- | --- | --- | --- | --- |
|  |  | **CTAB** | | | **GXT NA/Arrow** | | | **QIAamp DNA mini kit** | | |
|  |  | **Range** | **Mean** | **CV (%)** | **Range** | **Mean** | **CV (%)** | **Range** | **Mean** | **CV (%)** |
| (OTUs) | 1 | 3-19 | 13 | 33 | 7-39 | 21 | 44 | 26-38 | 32 | 11 |
|  | 2 | 7-18 | 15 | 26 | 13-32 | 21 | 27 | 24-38 | 31 | 13 |
|  | Both | 3-19 | 14 | 29 | 7-39 | 21 | 35 | 24-38 | 32 | 12 |
| Genera | 1 | 3-7 | 5.6 | 20 | 5-14 | 8.1 | 39 | 9-14 | 12 | 15 |
|  | 2 | 4-8 | 6.4 | 25 | 4-13 | 8.0 | 36 | 9-13 | 11 | 15 |
|  | Both | 3-8 | 6.0 | 24 | 4-14 | 8.1 | 36 | 9-14 | 12 | 15 |
| Shannon index | 1 | 0.763-2.11 | 1.8 | 24 | 1.25-2.46 | 2.0 | 17 | 2.23-2.39 | 2.3 | 2.5 |
|  | 2 | 1.24-2.24 | 1.9 | 16 | 1.72-2.38 | 2.0 | 9.1 | 2.13-2.50 | 2.3 | 5.3 |
|  | Both | 0.763-2.24 | 1.8 | 20 | 1.25-2.46 | 2.0 | 13 | 2.13-2.50 | 2.3 | 4.0 |
| Simpson index | 1 | 0.428-0.816 | 0.74 | 16 | 0.642-0.840 | 0.78 | 7.5 | 0.828-0.844 | 0.84 | 0.6 |
|  | 2 | 0.637-0.838 | 0.77 | 7.8 | 0.735-0.844 | 0.79 | 3.8 | 0.808-0.858 | 0.84 | 1.8 |
|  | Both | 0.428-0.838 | 0.76 | 12 | 0.642-0.844 | 0.79 | 5.8 | 0.808-0.858 | 0.84 | 1.3 |

Table S13

Effects of DNA extraction method, day of extraction and sputum sample on microbiome alpha diversity (Operational taxonomic units (OTUs), genera, Shannon indices and Simpson indices). *p*-values were calculated using Kruskal–Wallis t-test.

| Alpha diversity parameter | Examined effectors | p-value |
| --- | --- | --- |
| Richness OTUs | DNA extraction method | 2.44E-08 |
|  | Day of extraction | 0.9172 |
|  | Sputum sample (A, B, C) | 0.9873 |
| Richness genera | DNA extraction method | 3.59E-07 |
|  | Day of extraction | 0.8615 |
|  | Sputum sample (A, B, C) | 0.9191 |
| Shannon diversity index | DNA extraction method | 5.77E-07 |
|  | Day of extraction | 0.8288 |
|  | Sputum sample (A, B, C) | 0.8114 |
| Simpson diversity index | DNA extraction method | 2.74E-06 |
|  | Day of extraction | 0.7753 |
|  | Sputum sample (A, B, C) | 0.6989 |

Table S14

Statistical analysis of effects on microbime composition. R^2^ and p-values were calculated with PERMANOVA (permutational multivariate analysis of variance).

| Examined effectors | R^2^ | p-value |
| --- | --- | --- |
| DNA extraction method | 0.63247 | 0.000999 |
| Day of extraction | 0.00635 | 0.7453 |
| Sputum sample (A, B, C) | 0.02563 | 0.6084 |

Table S15

Statistical analysis of differences between different DNA extraction methods and their effects on microbiome alpha diversity (Operational taxonomic units (OTUs), genera, Shannon indices and Simpson indices). *p*-values were calculated using Kruskal–Wallis t-test and adjusted with Benjamini-Hochberg method used to control the false discovery rate.

| Alpha diversity parameter | Comparison | Z | P.unadj | P.adj |
| --- | --- | --- | --- | --- |
| Richness OTUs | CTAB - GXT NA/Arrow | -2.50382 | 1.23E-02 | 1.23E-02 |
|  | CTAB - QIAmp DNA mini kit | -5.89883 | 3.66E-09 | 1.10E-08 |
|  | GXT NA/Arrow - QIAmp DNA mini kit | -3.39501 | 6.86E-04 | 1.03E-03 |
| Richness genera | CTAB - GXT NA/Arrow | -2.08362 | 3.72E-02 | 3.72E-02 |
|  | CTAB - QIAmp DNA mini kit | -5.40139 | 6.61E-08 | 1.98E-07 |
|  | GXT NA/Arrow - QIAmp DNA mini kit | -3.31777 | 9.07E-04 | 1.36E-03 |
| Shannon diversity index | CTAB - GXT NA/Arrow | -1.39841 | 1.62E-01 | 1.62E-01 |
|  | CTAB - QIAmp DNA mini kit | -5.18048 | 2.21E-07 | 6.64E-07 |
|  | GXT NA/Arrow - QIAmp DNA mini kit | -3.78207 | 1.56E-04 | 2.33E-04 |
| Simpson diversity index | CTAB - GXT NA/Arrow | -0.94287 | 3.46E-01 | 3.46E-01 |
|  | CTAB - QIAmp DNA mini kit | -4.77791 | 1.77E-06 | 5.31E-06 |
|  | GXT NA/Arrow - QIAmp DNA mini kit | -3.83504 | 1.26E-04 | 1.88E-04 |

Table S16

List of bacterial genera and *p*-values calculated using Kruskal–Wallis t-tests for the significance of difference in proportion of each genus between samples extracted using different DNA extraction method. *, *p*-value <0.05; **, *p*-value <0.01; ***, *p*-value <0.001; NS, not significant (Kruskal-Wallis t-test).

| **Genus** | ***p*-value** | ***Significance*** |
| --- | --- | --- |
| Abiotrophia | 0.747883 | NS |
| Acinetobacter | 0.351501 | NS |
| Actinomyces | 1.46E-06 | *** |
| Aggregatibacter | 0.388896 | NS |
| Alloprevotella | 0.363974 | NS |
| Catonella | 0.007449 | ** |
| Eikenella | 0.607318 | NS |
| Fusobacterium | 0.086794 | NS |
| Granulicatella | 0.99509 | NS |
| Haemophilus | 0.0389 | * |
| Klebsiella | 0.357217 | NS |
| Lachnoanaerobaculum | 0.005422 | ** |
| Megasphaera | 0.083028 | NS |
| Neisseria | 0.000467 | *** |
| Oribacterium | 0.00403 | ** |
| Paraprevotella | 0.000945 | *** |
| Parvimonas | 2.48E-07 | *** |
| Pasteurella | 0.072304 | NS |
| Peptococcus | 0.388896 | NS |
| Porphyromonas | 0.354416 | NS |
| Prevotella | 5.84E-09 | *** |
| Pseudomonas | 0.388896 | NS |
| Staphylococcus | 0.028112 | * |
| Stomatobaculum | 0.019052 | * |
| Streptococcus | 1.01E-06 | *** |
| Tannerella | 0.607318 | NS |
| Veillonella | 1.25E-09 | *** |

Table S17

Average percentage of Gram-positive bacteria in samples extracted with different method.

| **DNA Extraction Method** | **Gram-positive %** |
| --- | --- |
| CTAB | 53.6% |
| GXT NA/Arrow | 45.7% |
| QIAamp DNA mini kit | 43.7% |

Table S18

Statistical analysis of differences between different DNA extraction methods and their effects on proportion of Gram-positive bacteria. *p*-values were calculated using Kruskal–Wallis t-test and adjusted with Benjamini-Hochberg method used to control the false discovery rate.

| Comparison | Z | P.unadj | P.adj |
| --- | --- | --- | --- |
| CTAB - GXT NA/Arrow | 3.938137 | 8.21E-05 | 1.23E-04 |
| CTAB - QIAmp DNA mini kit | 5.660717 | 1.51E-08 | 4.52E-08 |
| GXT NA/Arrow - QIAmp DNA mini kit | 1.666717 | 0.956E-02 | 9.56E-02 |

Table S19

Resistome diversity parameters of spiked sputum samples. Table shows minimal, maximal (range) and average (mean) number of detected amplicons, genes associated with antimicrobial resistance (AMR genes) and antimicrobial classes indices for three DNA extraction methods CTAB, GXT NA/Arrow and QIAamp DNA mini kit. Values are given for two separate days of extraction and for both days combined.

| **Parameter** | **Day** | **Data obtained according to DNA extraction method** | | | | | | | | |
| --- | --- | --- | --- | --- | --- | --- | --- | --- | --- | --- |
|  |  | **CTAB** | | | **GXT** | | | **QIAamp DNA mini kit** | | |
|  |  | **Range** | **Mean** | **CV (%)** | **Range** | **Mean** | **CV (%)** | **Range** | **Mean** | **CV (%)** |
| AMR gene amplicons | 1 | 0-25 | 19 | 39 | 8-33 | 22 | 30 | 24-43 | 33 | 15 |
|  | 2 | 19-27 | 22 | 11 | 20-39 | 24 | 24 | 25-37 | 32 | 13 |
|  | Both | 0-27 | 21 | 27 | 8-39 | 23 | 27 | 24-43 | 33 | 14 |
| AMR genes | 1 | 2-18 | 14 | 34 | 7-21 | 15 | 25 | 16-26 | 20 | 14 |
|  | 2 | 14-18 | 16 | 11 | 13-23 | 16 | 18 | 19-22 | 20 | 7 |
|  | Both | 2-18 | 15 | 24 | 7-23 | 15 | 22 | 16-26 | 20 | 11 |
| Antimicrobial classes | 1 | 2-7 | 5.6 | 27 | 3-7 | 5.6 | 22 | 6-8 | 6.8 | 12 |
|  | 2 | 5-7 | 6.1 | 13 | 5-7 | 5.8 | 14 | 6-8 | 6.7 | 11 |
|  | Both | 2-7 | 5.8 | 21 | 3-7 | 5.7 | 18 | 6-8 | 6.7 | 11 |

Table S20

Effects of DNA extraction method, day of extraction and sputum sample on resistome diversity (number of detected amplicons, genes associated with antimicrobial resistance (AMR genes) and antimicrobial classes). *p*-values were calculated using Kruskal–Wallis t-test.

| Resistome results | Examined effectors | *p*-value |
| --- | --- | --- |
| Amplicons | DNA extraction method | 5.94E-07 |
|  | Day of extraction | 0.6898 |
|  | Sputum sample (A, B, C) | 0.3504 |
| AMR genes | DNA extraction method | 1.17E-06 |
|  | Day of extraction | 0.6383 |
|  | Sputum sample (A, B, C) | 0.8424 |
| Antimicrobial classes | DNA extraction method | 0.00471 |
|  | Day of extraction | 0.7219 |
|  | Sputum sample (A, B, C) | 0.00058 |

Table S21

Statistical analysis of effects on resistome composition. R^2^ and *p*-values were calculated with PERMANOVA (permutational multivariate analysis of variance).

| **Examined effectors** | **R^2^** | ***p*-value** |
| --- | --- | --- |
| **DNA extraction method** | 0.5422 | 0.000999 |
| **Day of extraction** | 0.00063 | 0.998 |
| **Sputum sample (A, B, C)** | 0.04738 | 0.2857 |

Table S22

Statistical analysis of differences between different DNA extraction methods and their effects on resistome diversity (number of detected amplicons, genes associated with antimicrobial resistance (AMR genes) and antimicrobial classes). *p*-values were calculated using Kruskal–Wallis t-test and adjusted with Benjamini-Hochberg method used to control the false discovery rate.

| Resistome results | Comparison | Z | P.unadj | P.adj |
| --- | --- | --- | --- | --- |
| Amplicons | CTAB - GXT NA/Arrow | -0.82346 | 4.10E-01 | 4.10E-01 |
|  | CTAB - QIAmp DNA mini kit | -4.99387 | 5.92E-07 | 1.78E-06 |
|  | GXT NA/Arrow - QIAmp DNA mini kit | -4.17041 | 3.04E-05 | 4.56E-05 |
| AMR genes | CTAB - GXT NA/Arrow | 0.138586 | 8.90E-01 | 8.90E-01 |
|  | CTAB - QIAmp DNA mini kit | -4.45608 | 8.35E-06 | 1.25E-05 |
|  | GXT NA/Arrow - QIAmp DNA mini kit | -4.59466 | 4.33E-06 | 1.30E-05 |
| Antimicrobial classes | CTAB - GXT NA/Arrow | 0.754602 | 0.450488 | 0.450488 |
|  | CTAB - QIAmp DNA mini kit | -2.38119 | 0.017257 | 0.025885 |
|  | GXT NA/Arrow - QIAmp DNA mini kit | -3.13579 | 0.001714 | 0.005142 |

Table S23

List of AMR genes and *p*-values calculated using Kruskal–Wallis t-tests for the significance of difference in proportion of each gene between samples extracted using different DNA extraction method.

| AMR gene | *p*-value |
| --- | --- |
| erm(B) | 3.02E-09 |
| penA | 3.40E-08 |
| erm(F) | 1.30E-06 |
| macB | 2.66E-06 |
| msr(D)/mel | 6.72E-06 |
| pbp2a | 8.54E-06 |
| catB7 | 1.32E-05 |
| pbp3 | 1.58E-05 |
| aadK | 1.90E-05 |
| femA | 1.90E-05 |
| mef(A) | 3.59E-05 |
| tet(Q) | 3.63E-05 |
| tet(M) | 4.59E-05 |
| aph(3') iib | 0.000168 |
| van(Y) | 0.000238 |
| tet(W) | 0.000371 |
| qacA/B | 0.000535 |
| uppP | 0.001038 |
| ermC | 0.002453 |
| cat(S) | 0.003088 |
| cfxA | 0.012013 |
| eptA | 0.017957 |
| msr(SA) | 0.018956 |
| tet(B) | 0.022464 |
| aph(2') ia | 0.039457 |
| mecA | 0.040073 |

# **Figures**

Figure S1

Preparation of aliquots of spiked sputum samples. Sputum samples collected from 16 different patients were pooled together and homogenised after the addition of mucolytic reagent. Pooled and homogenised mixture of sputa was separated in in three aliquots A, B and C, which were then spiked with *Acinetobacter baumannii*, *Klebsiella pneumoniae* and *Pseudomonas aeruginosa*. All three bacteria were present all three spiked sputum samples in either high (5.5 ×10^5^ cells/mL), medium (5.5 ×10^4^ cells/mL) or low (5.5 ×10^3^ cells/mL) concentration. Spiked sputum samples A, B and C were then split into 200 µL aliquots.


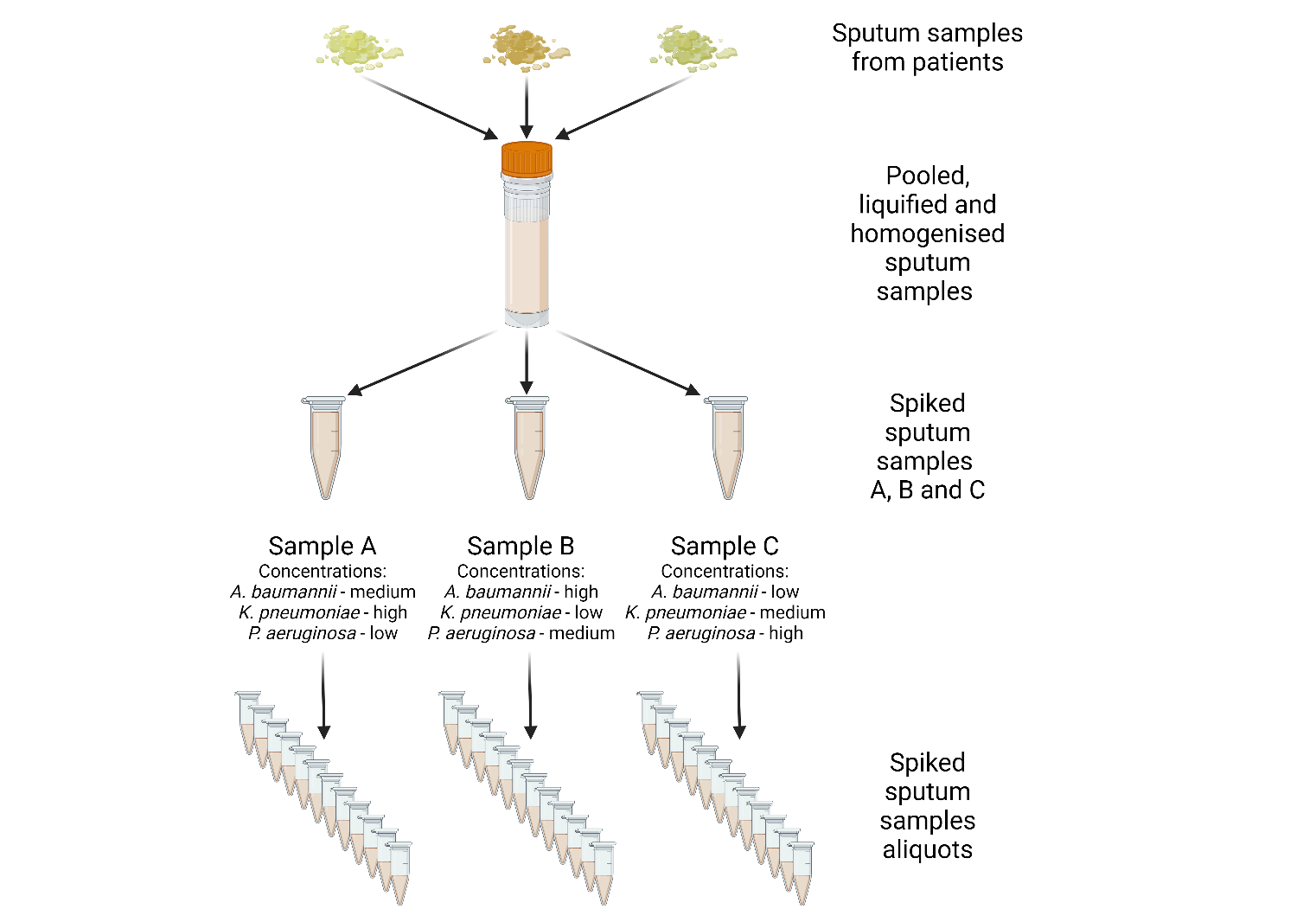


Figure S2

Extraction of DNA from spiked sputum samples. DNA was extracted from aliquots of all three sputum samples A, B and C using three different extraction methods: CTAB-based method, GXT NA kit with Arrow automated system and QIAamp DNA mini kit. Each DNA extraction method was performed on two separate days in three technical replicates on each day to examine repeatability of extractions.


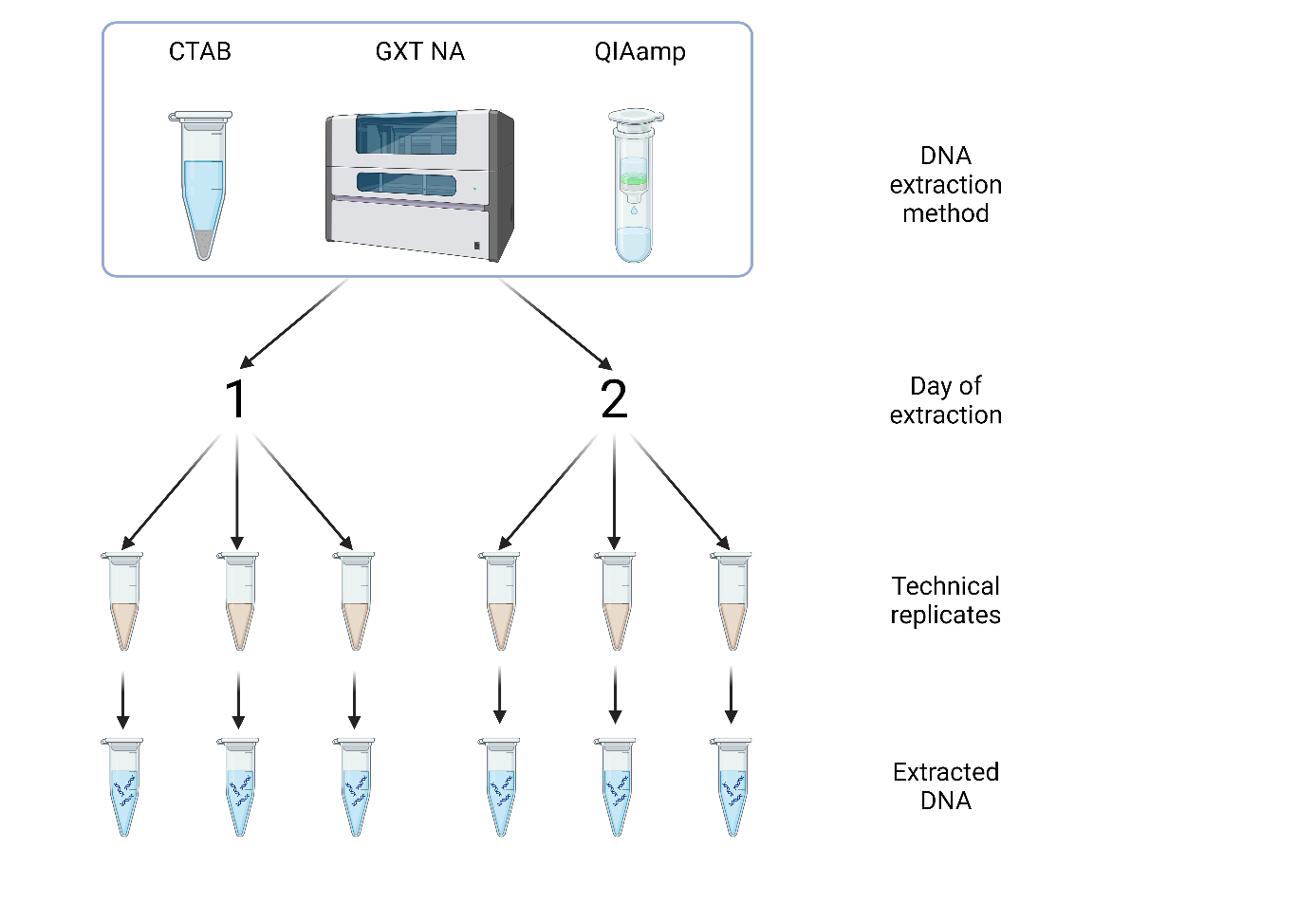


Figure S3

Comparison of yield and fragmentation for all three DNA extraction methods. (A) Yield of extracted DNA for each extraction method for each day. (B) Fragmentation of extracted DNA defined as the as Genomic Quality Score for each extraction method for each day. ***, *p*-value <0.001; (Kruskal-Wallis t-test).


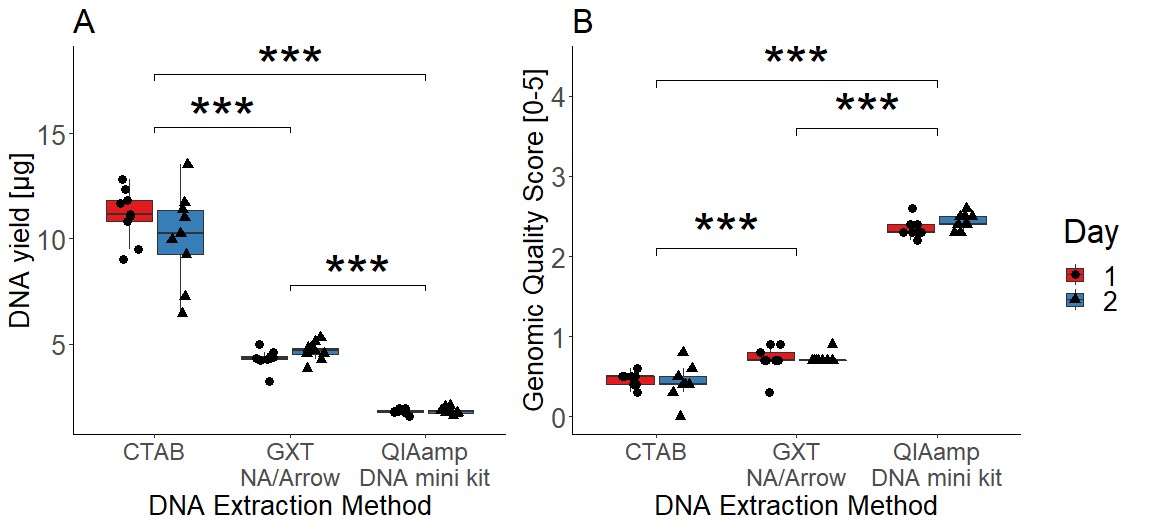


Figure S4

Numbers of amplicons (A), genes associated with antimicrobial resistance (AMR genes) (B), and antibiotic classes (C) which were detected in spiked sputum samples according to different DNA extraction methods. The boxplots show median values and quartiles for each DNA extraction method and day of extraction. *, p-value <0.05; **, p-value <0.01; ***, p-value <0.001; (Kruskal-Wallis t-test).


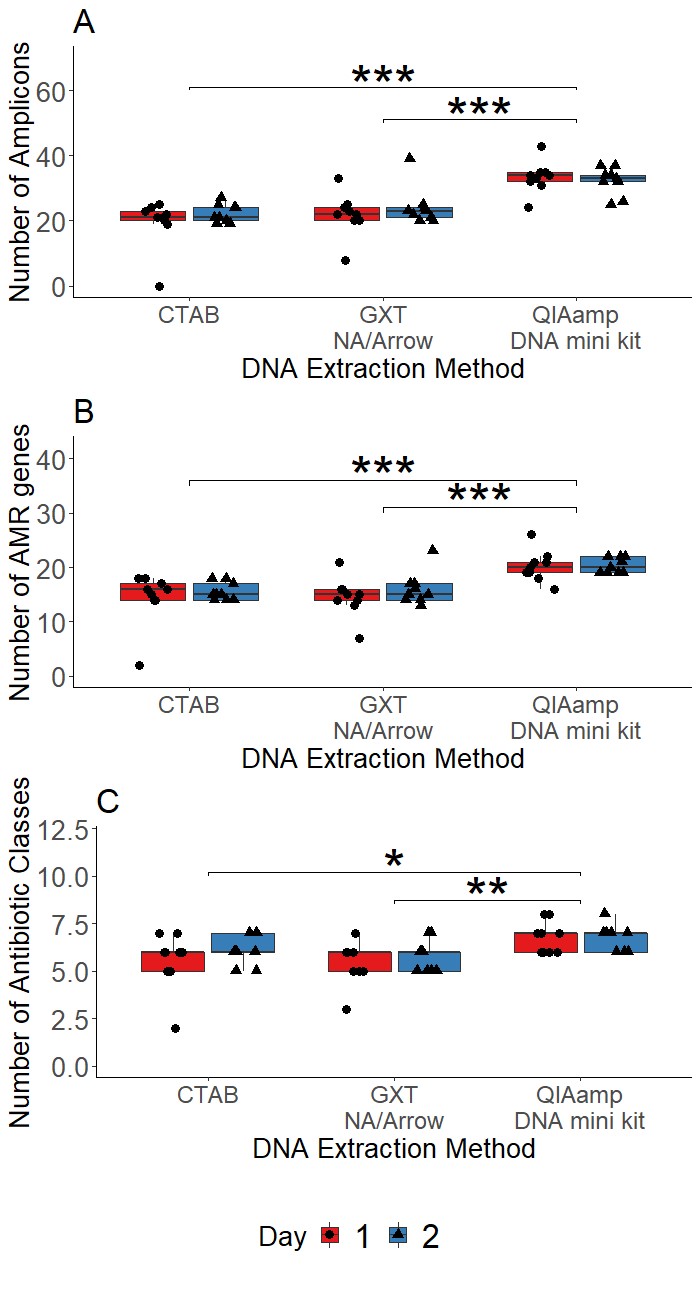

Supplement: Supplemental material — Supplemental figures and tables. [file msystems.00735-24-s0001.docx]
